# TERT rs10069690 variant is linked to reduced cholangiocarcinoma incidence but adverse prognosis in patients undergoing resection

**DOI:** 10.1136/egastro-2025-100320

**Published:** 2026-05-29

**Authors:** Isabella Lurje, Justus Pein, Paul Horn, Deniz Uluk, Marlene Kohlhepp, Minh Duc Phan, Saskia Niklisch, Frederik Schliephake, Niharika Jakhar, Kai Markus Schneider, Florian Roßner, David Horst, Johann Pratschke, Frank Tacke, Carolin Victoria Schneider, Georg Lurje

**Affiliations:** 1 Department of Hepatology and Gastroenterology, Charité - Universitätsmedizin Berlin, Berlin, Germany; 2 Department of General, Visceral and Transplantation Surgery, Heidelberg University Hospital, Heidelberg, Germany; 3 Department of Surgery, Charité - Universitätsmedizin Berlin, Berlin, Germany; 4 Berlin Institute of Health at Charité – Universitätsmedizin Berlin, BIH Biomedical Innovation Academy, BIH Charité Digital Clinician Scientist Program, Berlin, Germany; 5 Department of Medicine III, University Hospital RWTH Aachen, Aachen, Germany; 6 Department of Medicine I, University Hospital Carl-Gustav-Carus Dresden, Dresden, Germany; 7 Institute of Pathology, Charité - Universitätsmedizin Berlin, Berlin, Germany

**Keywords:** Cholangiocarcinoma, Polymorphism, Single Nucleotide, Telomerase, Prognosis, Proteomics

## Abstract

**Background:**

Cholangiocarcinoma (CCA) is a rare cancer, with limited understanding of genetic and prognostic determinants. We aimed to explore genetic risk factors, assess their prognostic implications and evaluate associated systemic and intratumoral features.

**Methods:**

We screened the UK Biobank to identify single-nucleotide polymorphisms (SNPs) potentially associated with intrahepatic CCA (International Classification of Diseases, 10th Revision (ICD-10) code: C22.1). Candidate SNPs were genotyped in a real-life cohort of 221 patients undergoing liver resection for intrahepatic or perihilar CCA at Charité – Universitätsmedizin Berlin. Intratumoral gene expression and pathway co-expression were examined. Serum proteomic profiles were evaluated in patients with intrahepatic CCA and the overall population.

**Results:**

In exploratory analyses, the telomerase reverse transcriptase *(TERT)* rs10069690 T allele was associated with a reduced risk of intrahepatic CCA (T allele vs C/C homozygotes: adjusted OR 0.824 (95% CI 0.713 to 0.951), p=0.008). However, T allele carriers undergoing liver resection for CCA had independently shorter overall survival (OS) compared with C/C homozygotes (median OS 21 (17–25) months vs 31 (24–38) months, p=0.034, HR 1.427 (1.023–1.991)). In serum proteomic analyses of the general population, presence of the T allele was associated with differences in immune-related pathways, including signatures consistent with increased lymphocyte differentiation and reduced NK-cell-mediated cytotoxicity. In intrahepatic CCA tumours, higher *TERT* mRNA expression was positively correlated with gene expression patterns consistent with increased cell cycle activity and regulatory T cell signatures, and negatively associated with pathways of cell differentiation, adhesion and immune effector function.

**Conclusions:**

These exploratory, hypothesis-generating findings suggest that the *TERT* rs10069690 variant may be associated with intrahepatic CCA risk and clinical outcomes, as well as with immune-related and proliferative pathways. The observed context-dependent associations warrant independent validation and further functional investigation.

**WHAT IS ALREADY KNOWN ON THIS TOPIC**

- Cholangiocarcinoma (CCA) is an aggressive malignancy that often arises in the absence of known risk factors and presents difficulties in prognostic risk stratification.

**WHAT THIS STUDY ADDS**

- The study reports an association between the telomerase reverse transcriptase (*TERT*) rs10069690 T allele and reduced risk of intrahepatic CCA, alongside an association with shorter overall survival in patients with established disease. The findings suggest a context-dependent role of *TERT* in CCA susceptibility and tumour progression, warranting independent validation.

**HOW THIS STUDY MIGHT AFFECT RESEARCH, PRACTICE OR POLICY**

- If confirmed, *TERT* genotyping could inform both risk assessment and prognosis in CCA and support future research into *TERT*-targeting.

**Figure:**
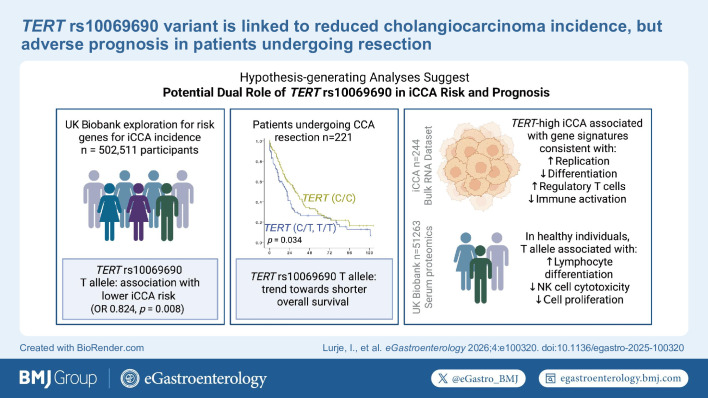
Visual Abstract Summarising the hypotheses generated in this exploratory study. Created in BioRender.com. CCA, cholangiocarcinoma; iCCA, intrahepatic CCA; NK, N-terminal kinase; *TERT*, telomerase reverse transcriptase.

## Introduction

 Although cholangiocarcinoma (CCA) accounts for only 15% of primary liver cancer cases in Western populations,^1^ it represents a challenging disease with often late diagnosis. While certain conditions, such as chronic biliary inflammatory diseases, markedly increase CCA risk, many cases arise sporadically without a clearly identifiable cause.^2 3^ Patients undergoing surgical resection experience high morbidity and mortality,^4^ with tumour recurrence rates exceeding 50%.^5 6^ Currently, risk stratification in patients with manifest disease focuses on tumour-specific factors, such as the mutational profile and the presence of lymphovascular or nodal invasion.^7^ Previous studies in hepatocellular carcinoma (HCC) have highlighted the role of single-nucleotide polymorphisms (SNPs) in influencing disease susceptibility (eg, in patatin-like phospholipase domain-containing protein 3 (*PNPLA3*)^8^) and prognosis (eg, in vascular endothelial growth factor (*VEGF*)^9^), but such evidence remains sparse in CCA.^10^

To address this discrepancy, we leveraged the UK Biobank (UKB), a population-based, prospective dataset of over 500 000 participants, to screen for SNPs associated with CCA. Among candidate variants, the telomerase reverse transcriptase (*TERT*) polymorphism rs10069690 emerged as a variant of interest for further investigation.

*TERT* is a catalytic component of the telomerase complex and is therefore central for telomere maintenance. Furthermore, TERT functions through a non-canonical pathway and increases the transcription of genes involved in proliferation, the cell cycle and, in the context of cancer, in epithelial-to-mesenchymal transition and invasiveness.^11^ The *TERT* rs10069690 variant has previously been associated with cancer susceptibility in multiple tumour types, including breast, ovarian and thyroid cancers.^12^ The rs10069690:T allele introduces an alternative splice site in *TERT*, promoting expression of a catalytically inactive isoform (INS1b) that may interfere with telomerase activity in a cell type-dependent manner.^13 14^

Given its established roles in genome stability and transcriptional regulation, the *TERT* rs10069690 variant represents a biologically plausible candidate for investigation in CCA, where its role remains poorly defined. The prognostic impact of the *TERT* variant was explored in patients undergoing liver resection for intrahepatic (iCCA) or perihilar CCA (pCCA). To gain insights into potential underlying mechanisms, we examined intratumoral gene expression profiles and serum proteomic data.

This work aims to improve the understanding of genetic factors contributing to CCA risk, prognosis and biology and to provide a foundation for future studies exploring diagnostic and therapeutic advances.

## Methods

### UKB screening and proteomics

The UKB is a population-based cohort study conducted in the UK from 2006 to 2010, enrolling 502 511 participants aged 37–73 years at baseline. Participants were registered with the UK National Health Service and provided informed consent for genotyping and data linkage to medical reports. Participants underwent initial examination, followed by extensive longitudinal follow-up. Blood samples and physical measurements were obtained at all visits.

We screened UKB data in October 2024, with data available until August 2023, investigating a panel of 26 SNP candidates for the risk of CCA. Polymorphisms were selected with a sufficient minor allele frequency (≥0.01) and based on their reported involvement in liver disease, particularly primary liver cancer. The candidate variants were selected from loci with established relevance to liver disease and that have been repeatedly evaluated in prior population-genetic association studies, including analyses in UKB. Therefore, we used UKB’s centrally processed genotypes data and imputed dosages without applying further study-specific quality control (QC) filters. Genotyping of the selected variants was available for a total of 4 75 089 participants in UKB. Candidate variants were obtained either from directly genotyped array data or from the UKB imputed-v3 resource.

Patients with International Classification of Diseases, 10th Revision (ICD-10) code C22.1 ‘intrahepatic bile duct carcinoma’ were identified. The study was conducted under UKB access number 71300.

The proteomic data were obtained using the Olink Proteomics platform (Olink Proteomics AB, Uppsala, Sweden), which quantifies the expression levels of targeted proteins. This platform employs proximity extension assay technology, allowing for the high-throughput analysis of multiple proteins simultaneously. The Olink Explore 3072 platform features 2941 immunoassays targeting 2925 proteins measured in 53 030 participants at baseline. Data are reported in Normalised Protein eXpression (NPX) units, measured on a log2 scale. The UKB plasma samples were processed at Olink’s Uppsala facilities (Sweden), using three NovaSeq 6000 Sequencing Systems (Illumina, San Diego, California, USA).

### Study population (Charité cohort)

A total of 221 consecutive patients who underwent liver resection for localised iCCA or pCCA at Charité – Universitätsmedizin Berlin between 2010 and 2020 were included in this study (‘Charité cohort’). The use of surgical samples and clinical data was approved by the local ethics committee (EA1/292/16 and EA1/111/21). Selected clinical and imaging characteristics of the cohort have been reported previously in relation to the role of body composition in iCCA.^15^ Preoperatively, cross-sectional imaging was used to exclude distant metastases and to visualise the extent of local invasion. The surgical approach involved hilar en bloc resection with radical lymphadenectomy and portal vein resection for pCCA. Surgical specimens underwent histopathological evaluation, including staging and grading, by a board-certified pathologist.

### Genotyping

Germline genomic DNA was extracted from tumour-free formalin-fixed paraffin-embedded (FFPE) tissue provided by the Department of Pathology at Charité – Universitätsmedizin Berlin. The tissue blocks were sectioned at 5 µm, with 5–6 sections collected in Eppendorf tubes (Eppendorf SE, Hamburg, Germany) and deparaffinised using xylene. DNA extraction was performed using the QIAamp DNA FFPE Tissue Kit (Qiagen N.V., Hilden, Germany), following the manufacturer’s instructions.

Genotyping for SNPs significantly linked to CCA risk was conducted with TaqMan real-time PCR assays (Applied Biosystems, Foster City, California, USA), according to the manufacturer’s protocol (online supplemental table 1). Briefly, sequences including the SNP of interest were amplified with forward and reverse primers on a QuantStudio 3 system (Applied Biosystems). Two minor groove binder probes with different reporter dyes visualised each allele. Genotypes were determined with the QuantStudio 3/5 Real-Time PCR Software (Version 2.8.0, Thermo Fisher Scientific, Waltham, Massachusetts, USA).

### Gene co-expression analysis and survival analysis in publicly available data

Gene expression in iCCA was studied in a published bulk RNA-sequencing dataset in Chinese, treatment-naïve patients undergoing surgical resection for iCCA.^16^ In 244 cases, information on intratumour *TERT* expression was available. Gene expression data and patient metadata were retrieved from the online supplemental materials of the publication by Dong *et al*.^16^ Gene co-expression analysis was performed using R Version 4.4.0. First, we calculated gene co-expression by performing Spearman rank correlation of all genes with *TERT*. P values were adjusted by Benjamini-Hochberg correction and rank scores were calculated as *rank* = –log *(padj) × rho*. Based on the ranked gene list, we performed gene set enrichment analysis (GSEA) on ‘biological process’ in the Gene Ontology (GO) database as implemented in the clusterProfiler Version 4.12.6 package and the org.Hs.eg.db Version 3.19.1 database with default settings.^17^ Redundant terms were removed by calling the “simplify” function from the clusterProfiler package with default settings.

GSEA results were imported into Cytoscape Version 3.10.3 and processed using the EnrichmentMap Version 3.5.0 pipeline.^18^ We generated a network of connected functional terms for all enriched terms with an adjusted p value<0.05. An edge cut-off of 0.375 was used to define edges, that is, connections between nodes, in the network. Subsequently, the network was clustered and annotated using AutoAnnotate Version 3.9.1.^19^ Annotations were manually adjusted to generate meaningful descriptions of the individual clusters, and legends were added with Legend creator (Version 1.1.7).

Survival analysis was performed using the cutpointr Version 1.1.2^20^ and survival Version 3.6–4 packages in R Version 4.4.0. The optimal gene expression cut-off was calculated as the maximum Youden index for overall survival (OS) status at 2 years. Kaplan–Meier estimates and log-rank tests were performed using the “survfit” and “survdiff” functions for univariable survival analysis with the dichotomised gene expression as the independent variable, while multivariable Cox proportional hazards models were built using the “coxph” function.

### Statistical analysis

Continuous variables are reported as median and IQR, categorical variables are reported as relative frequencies (%). Binomial logistic regression was conducted to assess the risk of disease, with results reported as ORs and 95% CIs. Postoperative survival was analysed with the log-rank method and visualised with Kaplan–Meier curves. Cox proportional hazards models were built for univariable and multivariable analyses of postoperative outcomes. For each SNP in UKB, we fitted logistic regression models with C22.1 case status as the dependent variable and genotype coded as effect-allele count (0/1/2). The prespecified covariate set was age at baseline (UKB field 21003–0.0), sex (31–0.0), body mass index (21001–0.0) and genetic principal components PC1–PC10 (22009–0.1 to 22009–0.10). Population structure was controlled by including PC1–PC10 in all models. Multivariable analysis for the Charité cohort included clinicopathological factors significant in univariable analysis while excluding factors with assumed collinearity. Survival outcomes was reported as recurrence-free survival (RFS), spanning the time from surgery until first documented recurrence, cancer-specific survival (CSS), encompassing the time from surgery until death from iCCA recurrence with censoring of all other causes of death and OS.

For proteomics analyses, Bonferroni correction was applied to adjust for multiple testing. Proteomics data were visualised as volcano plots. GO analysis was performed with Enrichr selecting GO Biological Process 2023 terminology with the Appyters extension.^21^ The remaining analyses were computed and visualised with SPSS Statistics Version 24 (IBM Corp., Armonk, New York, USA).

### Data and code availability

The code for the UKB analyses is available on request and will be uploaded to the UKB repository together with the dataset and can be accessed via UKB. The code for gene co-expression and survival analyses from public bulk RNAseq data is available online (https://github.com/hornp89/TERT_iCCA_RNAseq).

## Results

### *TERT* rs10069690:T is associated with decreased incidence of iCCA

**Figure 1:**
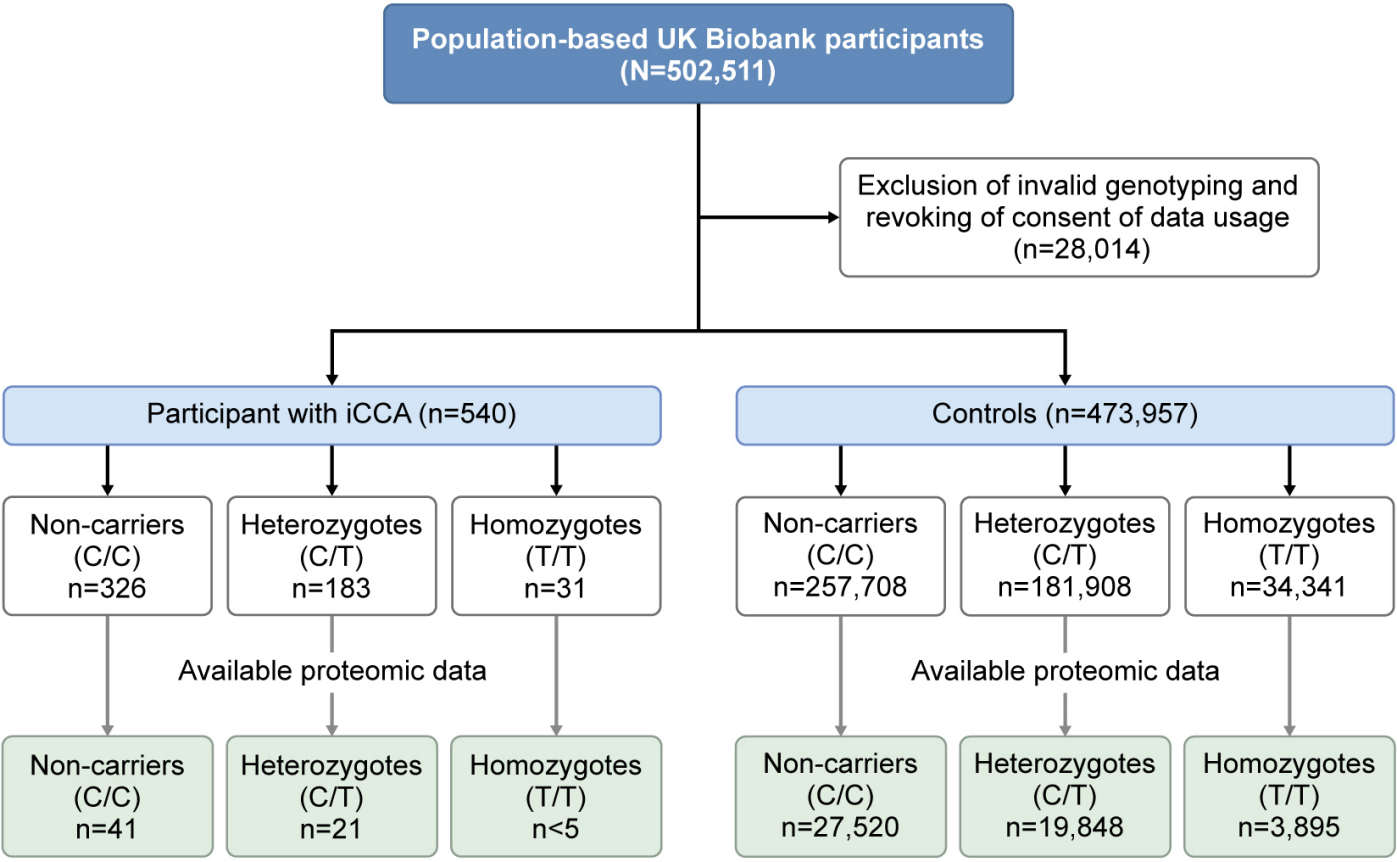
Overview of the analysed cohorts in the UK Biobank. n<5, masked for privacy. Participants with iCCA were identified with the ICD-10 code C22.1. iCCA, intrahepatic cholangiocarcinoma; ICD, International Classification of Diseases.

We analysed the association between a panel of 26 SNPs and diagnoses of ICD-10 code C22.1 ‘intrahepatic bile duct carcinoma’. A total of 540 individuals diagnosed with iCCA using ICD-10 code C22.1 were identified (figure 1). The risk of iCCA (C22.1) was significantly reduced only in carriers of *TERT* rs10069690:T (T allele vs C/C homozygotes: adjusted OR (95%CI): 0.824 (0.713 to 0.951), p=0.008, table 1).

**Table 1:** Screening of gene variants for association with iCCA in UK Biobank

Gene variant			Association with iCCA (ICD-10 code C22.1)	
Gene	rsID	Risk allele	OR (95% CI)	P value
*TERT*	rs10069690	T	0.824 (0.713 to 0.951)	**0.008**
*HSD17B13*	rs72613567	TA	0.922 (0.804 to 1.058)	0.249
*APOE*	rs2229426	A	1.333 (0.811 to 2.191)	0.256
*HLA-DPA1*	rs3077	G	1.088 (0.936 to 1.265)	0.273
*IL-6*	rs1800795	C	1.069 (0.946 to 1.209)	0.287
*SLC22A4*	rs142371324	T	0.489 (0.122 to 1.962)	0.313
*HLA-DPB1*	rs9277535	G	1.059 (0.924 to 1.215)	0.410
*PNPLA3*	rs738409	G	1.059 (0.918 to 1.221)	0.433
*HLA*	rs2856718	T	1.038 (0.917 to 1.176)	0.555
*CD3EAP, ERCC1*	rs3212986	A	0.960 (0.834 to 1.104)	0.562
*MTARC1*	rs2642438	A	0.969 (0.849 to 1.106)	0.640
*NKG2D*	rs2617167	A	0.966 (0.836 to 1.116)	0.641
*HLA-DQB2*	rs7453920	A	0.973 (0.861 to 1.098)	0.656
*TNF*	rs1800629	A	1.035 (0.890 to 1.204)	0.658
*RPS6KA3*	rs4444903	G	0.971 (0.847 to 1.113)	0.674
*CXCL1*	rs4073	A	0.977 (0.865 to 1.102)	0.700
*TM6SF2*	rs58542926	T	1.037 (0.828 to 1.297)	0.754
*GSTO1*	rs4925	A	0.982 (0.861 to 1.119)	0.784
*MST1*	rs3197999	A	1.018 (0.891 to 1.164)	0.791
*HLA-DQA1*	rs9272105	A	0.986 (0.875 to 1.112)	0.822
*MTHFR*	rs1801131	G	0.986 (0.866 to 1.123)	0.829
*ABCC2*	rs3740066	T	1.011 (0.894 to 1.144)	0.862
*INSR*	rs780094	T	0.992 (0.876 to 1.122)	0.893
*ADH1B*	rs455804	A	1.005 (0.868 to 1.165)	0.945
*LDLR*	rs137853964*	A	8.68E-05 (2.9E-128 to 2.597E+119)	0.949
*GCKR*	rs1260326	T	0.996 (0.881 to 1.127)	0.952

* The low frequency of the rs137853964 variant results in limited statistical power and wide CIs; estimates should therefore be interpreted with caution.

CI, confidence interval; iCCA, intrahepatic cholangiocarcinoma; ICD, International Classification of Diseases; OR, odds ratio.

### *TERT* rs10069690:T is independently associated with poor OS after CCA resection

A total of 221 patients underwent liver resection for CCA between February 2010 and December 2020. Of these, 130 patients were diagnosed with iCCA and 91 patients with pCCA. Patient characteristics were reported in table 2. Median follow-up after resection was 17 months (range: 0–150 moths). During this period, 89 (40.3%) patients experienced recurrence and 144 (65.2%) died during the observation period.

**Table 2:** Patient characteristics in the Charité CCA cohort

	iCCA n (%) (n=130)	pCCA n (%) (n=91)	Total n (%) (n=221)
Patient demographics			
Sex			
Female	54 (41.5)	32 (35.2)	86 (38.9)
Male	76 (58.5)	59 (64.8)	135 (61.1)
Median age (IQR)	66.2 (58.0–73.0)	66.5 (56.6–73.2)	66.4 (57.7–73.2)
Mean BMI (IQR)	25.6 (22.9–28.5)	25.0 (22.7–28.4)	25.4 (13.4–40.5)
Tumour characteristics			
Tumour stage UICC			
I	45 (34.6)	6 (6.6)	51 (23.1)
II	38 (29.2)	35 (38.5)	73 (33.0)
III	35 (26.9)	44 (48.3)	79 (35.7)
IV	12 (9.2)	6 (6.6)	18 (8.1)
Tumour grading			
G1	13 (10.0)	9 (9.9)	22 (9.9)
G2	84 (64.6)	58 (63.7)	142 (64.3)
G3	33 (25.4)	24 (26.4)	57 (25.8)
Resection status			
R0	91 (70.0)	66 (72.5)	157 (71.0)
R1/Rx	39 (30.0)	25 (27.5)	64 (29.0)
pT category			
T1-2	97 (74.6)	72 (79.1)	169 (76.5)
T3-4	33 (25.4)	19 (20.9)	52 (23.5)
pN category			
N0	89 (68.5)	44 (48.4)	133 (60.2)
N1	41 (31.5)	47 (51.6)	88 (39.9)
pM category			
M0	113 (86.9)	85 (93.4)	198 (89.6)
M1	17 (13.1)	6 (6.6)	23 (10.4)
Treatment			
Type of resection			
Bisegmentectomy	9 (6.9)	1 (1.1)	10 (4.5)
Right hemihepatectomy	31 (23.8)	2 (2.2)	33 (14.9)
Extended right hemihepatectomy	38 (29.3)	37 (40.6)	75 (33.9)
Left hemihepatectomy	25 (19.2)	8 (8.8)	33 (14.9)
Extended left hemihepatectomy	22 (16.9)	40 (44.0)	62 (28.1)
Atypical resection	3 (2.3)	0 (0.0)	3 (1.4)
Other	2 (1.6)	3 (3.3)	5 (2.3)
Neoadjuvant chemotherapy			
No	117 (90.0)	89 (97.8)	206 (93.2)
Yes	13 (10.0)	2 (2.2)	15 (6.8)
Blood transfusions			
No	90 (69.2)	75 (82.4)	165 (74.7)
Yes	40 (30.8)	16 (17.6)	56 (25.3)
FFP transfusions			
No	64 (49.3)	72 (79.1)	136 (62.0)
Yes	66 (50.8)	19 (20.9)	85 (38.5)

FFP, fresh frozen plasma; iCCA, intrahepatic cholangiocarcinoma; pCCA, perihilar cholangiocarcinoma; pM, pathological distant metastasis status; pN, pathological lymph node status; UICC, Union for International Cancer Control.

We tested the *TERT* rs10069690 polymorphism due to its association with C22.1, as well as four additional top genes for their association with clinical characteristics and long-term prognosis (online supplemental table 2). A total of 135 patients with CCA were homozygous (C/C) for *TERT* rs10069690, while 85 individuals harboured the T allele (68 heterozygous, 17 homozygous), genotyping failed in one (0.4%) sample. Baseline patient and tumour characteristics between *TERT* rs10069690 C/C homozygotes and T allele carriers showed no significant differences, apart from higher preoperative CA19-9 levels in T allele carriers with iCCA (online supplemental table 3).

**Figure 2:**
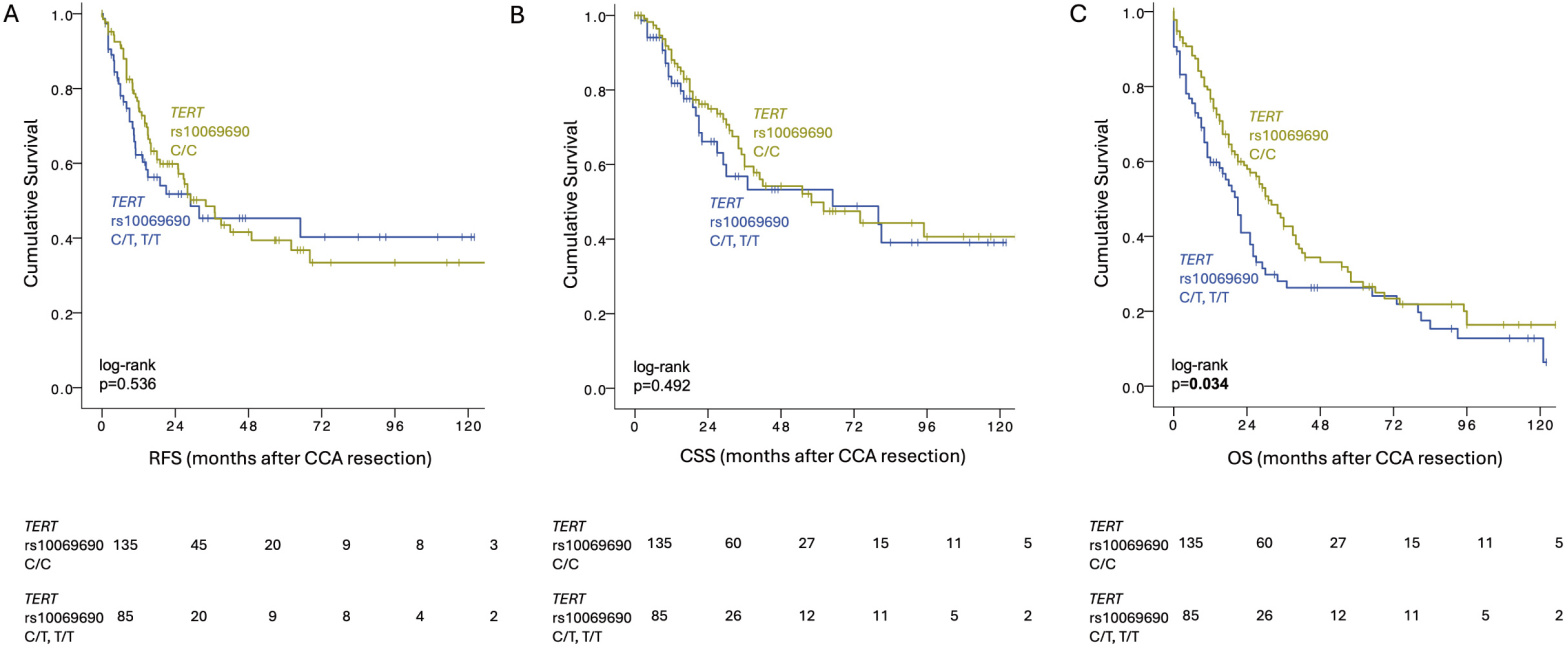
Postoperative survival by *TERT* rs10069690 genotype. (**A**) RFS, (**B**) CSS, (**C**) OS after CCA resection. CCA, cholangiocarcinoma; CSS, cancer-specific survival; OS, overall survival; RFS, recurrence-free survival; recurrence-free survival; *TERT*, telomerase reverse transcriptase.

Of the five tested polymorphisms, only *TERT* rs10069690 was associated with postoperative outcomes in the overall CCA cohort. The *TERT* rs10069690 genotype did not significantly impact RFS (T allele vs C/C homozygotes: 34 (23–45) months vs 29 (0–72) months, p=0.536, figure 2A) and CSS (T allele vs C/C homozygotes: 65 (1–129) months vs 58 (24–92) months, p=0.492, figure 2B). However, patients with CCA homozygous for the C allele had significantly longer OS than carriers of the *TERT* rs10069690:T (T allele vs C/C homozygotes: median OS 21 (17–25) months vs 31 (24–38) months, HR (95%CI): 1.427 (1.023 to 1.991), p=0.034, figure 2C). In the iCCA subgroup, C/C homozygotes had a non-significant trend towards longer OS, when compared with individuals with a T allele (T allele vs C/C homozygotes: median OS 18 (11–25) months vs 31 (21–41) months, HR (95%CI): 1.268 (0.821 to 1.957), p=0.278). Similarly, patients with pCCA and homozygous for the C allele had a non-significant trend towards longer OS (T allele vs C/C homozygotes: 32 (24–40) months vs 21 (19–23) months, HR (95%CI): 1.527 (0.899 to 2.594), p=0.110, online supplemental figure 1, online supplemental table 4). Furthermore, in the pCCA subgroup, patients with a *TM6SF2* rs58542926 CT or TT genotype had significantly shorter CSS and OS than patients with a homozygous (C/C) genotype (CSS: 24 (16–32) months vs 74 (2–146) months, p=0.018; OS 18 (14–22) months vs 29 (21–37) months, p=0.022).

We analysed clinicopathological factors correlating with prognosis and found that in the pooled cohort, Union for International Cancer Control (UICC) tumour stage, resection status, nodal positivity, lymphovascular invasion and vascular invasion were associated with RFS, CSS and OS, while perineural invasion was solely associated with decreased RFS and CSS. Higher patient age (>65 years) was associated with longer RFS (table 3). Separating the analysis by iCCA (online supplemental table 5) and pCCA (online supplemental table 6) demonstrated directionally similar prognostic trends but, likely, due to smaller sample size, only nodal positivity and lymphovascular invasion remained significant in iCCA, while in pCCA, UICC tumour stage, resection status, pathological tumour (pT) category, nodal positivity, vascular invasion and perineural invasion were associated with OS.

**Table 3:** Patient and tumour characteristics in association with long-term outcomes

	Recurrence-free survival		Cancer-specific survival		Overall survival	
	Median months (95% CI)	HR (95% CI)	Median months (95% CI)	HR (95% CI)	Median months (95% CI)	HR (95% CI)
Sex						
Female	37 (0 to 80)	1 (ref)	95 (n.a.)	1 (ref)	26 (18 to 35)	1.253 (0.893 to 1.757)
Male	27 (11 to 42)	1.445 (0.934 to 2.235)	41 (18 to 64)	1.496 (0.904 to 2.475)	25 (19 to 32)	1 (ref)
Log-rank p value	0.095		0.113		0.186	
Age in years						
≤65	19 (7 to 31)	1 (ref)	55 (27 to 83)	1 (ref)	30 (21 to 39)	1 (ref)
>65	39 (n.a.)	0.587 (0.387 to 0.892)	81 (22 to 140)	0.797 (0.495 to 1.282)	21 (15 to 27)	1.211 (0.865 to 1.694)
Log-rank p value	**0.011**		0.508		0.129	
BMI, kg/m^2^						
≤25	15 (0 to 35)	1 (ref)	80 (52 to 108)	1 (ref)	22 (15 to 29)	1 (ref)
>25	32 (17 to 47)	0.789 (0.515 to 1.208)	42 (12 to 72)	1.023 (0.628 to 1.666)	26 (19 to 33)	0.946 (0.677 to 1.322)
Log-rank p value	0.271		0.809		0.936	
Tumour stage						
UICC I and II	62 (24 to 100)	1 (ref)	81 (n.a.)	1 (ref)	34 (24 to 44)	1 (ref)
UICC III and IV	16 (11 to 21)	1.816 (1.181 to 2.794)	35 (30 to 41)	1.793 (1.099 to 2.925)	21 (16 to 26)	1.534 (1.097 to 2.144)
Log-rank p value	**0.006**		**0.017**		**0.011**	
Tumour grading						
G1 and G2	36 (24 to 50)	1 (ref)	65 (29 to 101)	1 (ref)	27 (20 to 34)	1 (ref)
G3	15 (12 to 18)	1.416 (0.904 to 2.220)	32 (n.a.)	1.201 (0.705 to 2.046)	21 (6 to 36)	1.191 (0.828 to 1.714)
Log-rank p value	0.124		0.542		0.356	
Resection status						
R0	34 (2 to 66)	1 (ref)	n.a.	1 (ref)	27 (20 to 34)	1 (ref)
R1/Rx	16 (1 to 31)	1.640 (1.053 to 2.555)	39 (27 to 51)	1.804 (1.095 to 2.973)	19 (9 to 29)	1.625 (1.145 to 2.307)
Log-rank p value	**0.026**		**0.018**		**0.006**	
pT category						
T1–2	42 (0 to 85)	1 (ref)	80 (53 to 107)	1 (ref)	28 (20 to 36)	1 (ref)
T3–4	19 (9 to 29)	1.312 (0.857 to 2.007)	34 (27 to 42)	1.892 (1.156 to 3.096)	19 (12 to 26)	1.512 (1.078 to 2.122)
Log-rank p value	0.207		0.147		0.092	
pN category						
N0	65 (n.a.)	1 (ref)	n.a.	1 (ref)	39 (20 to 58)	1 (ref)
N1	15 (8 to 22)	2.260 (1.430 to 3.571)	30 (16 to 44)	3.166 (1.855 to 5.404)	16 (11 to 21)	2.583 (1.781 to 3.748)
Log-rank p value	**<0.001**		**<0.001**		**<0.001**	
Lymphovascular invasion						
L0	49 (n.a.)	1 (ref)	80 (n.a.)	1 (ref)	27 (19 to 35)	1 (ref)
L1	13 (8 to 18)	2.414 (1.536 to 3.795)	31 (23 to 39)	2.745 (1.626 to 4.633)	21 (9 to 33)	1.710 (1.179 to 2.479)
Log-rank p value	**<0.001**		**0.004**		**0.043**	
Vascular invasion						
V0	37 (11 to 63)	1 (ref)	n.a.	1 (ref)	34 (25 to 43)	1 (ref)
V1	13 (7 to 20)	1.965 (1.203 to 3.211)	27 (15 to 40)	2.220 (1.274 to 3.868)	18 (14 to 22)	1.530 (1.006 to 2.327)
Log-rank p value	**0.006**		**<0.001**		**0.004**	
Perineural invasion						
Pn0	49 (14 to 84)	1 (ref)	95 (n.a.)	1 (ref)	27 (17 to 37)	1 (ref)
Pn1	16 (12 to 20)	1.723 (1.130 to 2.627)	34 (29 to 39)	1.795 (1.110 to 2.901)	21 (14 to 28)	1.299 (0.926 to 1.823)
Log-rank p value	**0.010**		**0.015**		0.125	

Median recurrence-free survival, cancer-specific survival and overall survival were estimated and reported with 95% CIs. n.a., estimates/ median not reached. HRs were reported with 95% CIs.

CI, confidence interval; HR, hazard ratio; pM, pathological distant metastasis status; pN, pathological lymph node status; pT, pathological tumour; UICC, Union for International Cancer Control.

The prognostic independence of the *TERT* polymorphism was tested together with significant variables from the univariable analysis, confirming *TERT* rs10069690 genotype (C/T heterozygotes or T/T homozygotes vs C/C homozygotes HR (95%CI): 2.088 (1.354 to 3.204), p=0.001), lymph node invasion (N1 vs N0 status HR (95%CI): 2.894 (1.419 to 5.903), p=0.003) and resection status (R1/Rx vs R0 HR (95%CI): 1.764 (1.109 to 2.804), p=0.016) as independently prognostic for OS in the overall Charité cohort (online supplemental table 7). Even though significance was not reached in univariable analysis when splitting the analysis by CCA subtype (online supplemental table 4), presence of a *TERT* T allele (C/T and T/T genotypes) remained significantly associated with inferior OS in multivariable analysis in iCCA (HR (95%CI): 2.219 (1.223 to 4.024), adjusted p=0.009), together with resection status (adjusted p=0.044, online supplemental table 8). Similarly, in pCCA, *TERT* genotype was determined as independently prognostic in multivariable analysis (HR (95%CI): 1.954 (1.006 to 3.794), adjusted p=0.048, online supplemental table 9). Patients with pCCA and the *TM6SF2* rs58542926 C/T or T/T genotype had significantly shorter OS than patients with a homozygous genotype (C/C), (HR (95%CI): 2.462 (1.182 to 5.129), adjusted p=0.016, online supplemental table 10).

### *TERT*-high tumours have a trend towards inferior OS

**Figure 3:**
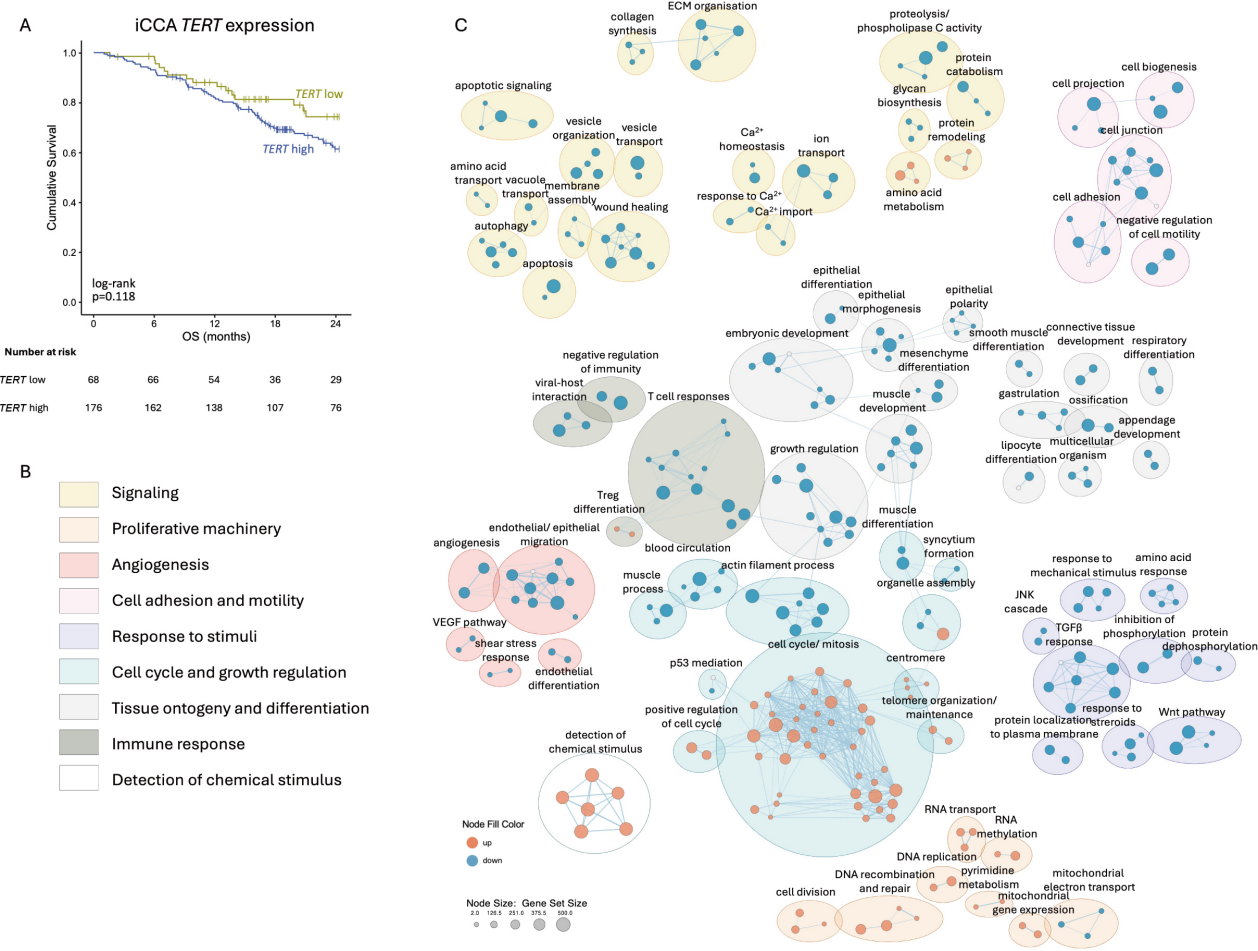
Role of intratumour *TERT* expression. (**A**) OS by *TERT* expression. (**B**) Main gene sets regulated under differential *TERT* expression. (**C**) Enrichment map of gene sets significantly correlated with *TERT* expression within the tumour microenvironment of iCCA. Node size represents the size of each gene set and connecting lines (edges) indicate the similarity of gene sets. ECM, extracellular matrix; iCCA, intrahepatic cholangiocarcinoma; JNK, c-Jun N-terminal kinase; OS, overall survival; *TERT*, telomerase reverse transcriptase; TGF, transforming growth factor; Treg, regulatory T cell; VEGF, vascular endothelial growth factor.

We analysed the impact of *TERT* expression on patient prognosis in the iCCA RNA sequencing dataset by Dong *et al*^16^ The optimised gene expression cut-off for OS status at 2 years was determined at 0.128 transcripts per million, identifying 68 patients with low and 176 patients with high *TERT* expression. A total of 15 of 68 (22.1%) patients with *TERT*-low tumours died during the follow-up period, while 62 of 176 (35.2%) patients with *TERT*-high tumours died (log-rank p=0.118, figure 3A). Multivariable Cox proportional hazard analysis included the parameters age, sex, tumour, node and metastases (TNM) stage and *TERT* expression and identified age and TNM stage as significant prognostically independent variables (online supplemental table 11).

Next, we investigated the co-expression of genes within the tumour microenvironment of iCCA in relation to *TERT* expression. Higher *TERT* expression in tumours was associated with 390 differentially regulated gene sets. The largest cohesive network comprised 39 upregulated gene sets pertaining to cell cycle/mitosis (figure 3B,C, online supplemental figure 2). Within this network, top enriched gene sets were related to the GO terms ‘regulation of mitotic cell cycle’, ‘mitotic cell cycle phase transition’, ‘nuclear division’ and ‘organelle fission*’* (online supplemental figure 2D). Further upregulated gene sets outside this network were annotated with DNA recombination and repair, DNA metabolism, pyrimidine metabolism and RNA transport and methylation (figure 3B,C).

A total of 18 gene sets pertaining to early and late cell differentiation, such as ‘epithelial polarity’, ‘mesenchyme differentiation’ and ‘gastrulation*’* were negatively associated with *TERT* expression. We also observed a downregulation of gene sets related to immune responses, especially T cell responses, with the immunosuppressive regulatory T cell (Treg) cluster as the only upregulated immune cluster (figure 3B,C). Gene sets annotated with cell adhesion, cell-cell junctions and cell immobility (‘negative regulation of cell motility*’*) were significantly downregulated in iCCAs with high *TERT* expression (online supplemental figure 2C).

### *TERT* rs10069690 T genotype is associated with systemically regulated immune pathways

**Figure 4:**
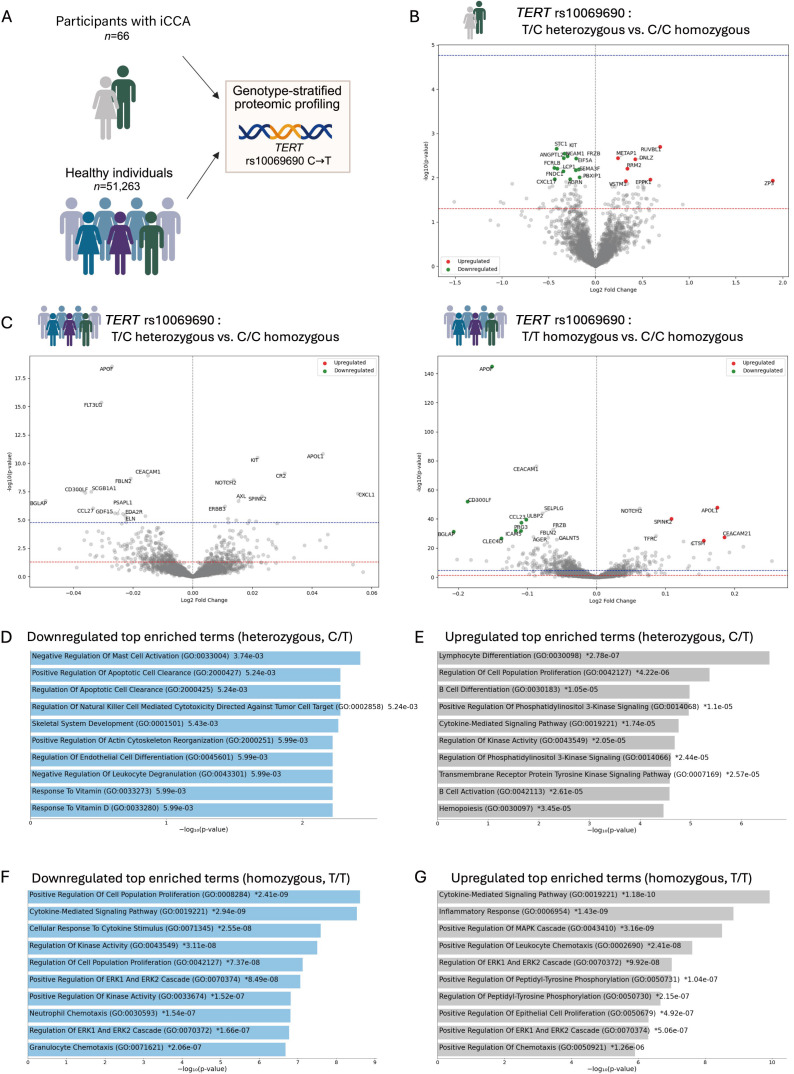
Proteomic profiling of *TERT* rs10069690:T carriers in pre-CCA and healthy populations. (**A**) Genotype-specific proteomic profiling was conducted in UK Biobank participants with ICD-10 code C22.1 and in healthy individuals. Volcano plots of alternatively regulated genes in (**B**) heterozygous (C/T) individuals with ICD-10 code C22.1 (intrahepatic CCA), (**C**) heterozygous (C/T) and homozygous (T/T) individuals from the control group, compared with homozygous for the C allele (C/C). Top 10 enriched annotated GO terms of downregulated genes in (**D**) heterozygous (C/T) and (F) homozygous (T/T) individuals and of upregulated genes in (**E**) heterozygous (C/T) and (**G**) homozygous (T/T) individuals. Created in BioRender.com. Hammerich, L (2026) (https://BioRender.com/80v5hxi). CCA, cholangiocarcinoma; GO, gene ontology; ICD, International Classification of Diseases; *TERT*, telomerase reverse transcriptase.

To assess systemic effects of the *TERT* rs10069690 polymorphism, we analysed serum proteomics data from UKB, stratified by *TERT* rs10069690 genotype (figure 4A). Proteomics data from 66 individuals who subsequently developed iCCA (C22.1) were analysed in an exploratory manner to investigate potential causative pathways associated with disease onset. However, none of the differentially expressed proteins reached statistical significance, likely due to the limited sample size in the genotype groups (figure 4B, group overview in figure 1).

Subsequently, we investigated serum proteomics in 51 263 individuals from the control population to examine whether the *TERT* rs10069690 polymorphism was associated with systemic responses in individuals without CCA. A total of 19 848 heterozygous individuals (C/T) and 3895 homozygous individuals (T/T) were compared with 27 520 homozygous individuals (C/C) with available proteomic data. In both heterozygous and homozygous T allele carriers, BGLAP, CD300LF and APOF were among the top 15 downregulated proteins, while CXCL1, APOL1 and SPINK2 were among the top 15 proteins with higher expression in T allele carriers compared with C/C homozygotes (figure 4C).

In heterozygous individuals, the downregulated top enriched terms were associated with ‘negative regulation of mast cell activation’, ‘apoptotic cell clearance’ and ‘regulation of natural killer cell-mediated cytotoxicity directed against tumour cell target’ (figure 4D). Upregulated top enriched terms were ‘lymphocyte differentiation’, ‘regulation of cell population proliferation’ and ‘B cell differentiation*’* (figure 4E, online supplemental table 12).

Despite the smaller sample size in the homozygous group, statistical differences in protein expression were more pronounced in this group, suggesting an additive allelic effect (figure 4C). The top downregulated enriched terms were ‘positive regulation of cell population proliferation’, ‘cytokine-mediated signalling pathway’ and ‘cellular response to cytokine stimulus’ (figure 4F). Upregulated top enriched terms included ‘cytokine-mediated signalling pathway’, ‘inflammatory response’ and ‘positive regulation of MAPK cascade*’* (figure 4G, online supplemental table 13).

## Discussion

Our study screened SNPs associated with CCA risk in the general population and identified the non-coding *TERT* rs10069690 polymorphism as being associated with both risk and prognosis of CCA. We found the *TERT* rs10069690:T SNP to be associated with reduced susceptibility to iCCA susceptibility in a large cohort of European individuals. Notably, carriers of the T allele had poorer OS after resection, while high *TERT* expression was associated with gene expression hallmarks of aggressive tumour biology.

*TERT* encodes the catalytic subunit of telomerase, with central functions in telomere maintenance—a process that counteracts replicative senescence due to telomere shortening during cell division.^22^ In most differentiated tissues, telomerase is inactive, leading to progressive telomere shortening and replicative senescence.^22^,^24^ Beyond this canonical, catalytic function, *TERT* extensively regulates transcriptional activity, cell cycle and apoptosis.^11 25 26^

The reactivation of *TERT* expression through gain-of-function mutations in its promoter is a common event in the malignant transformation of various human cancers.^27 28^ Particularly in HCC this is one of the most prevalent and early genetic alterations in carcinogenesis, with over 60% of HCC cases harbouring *TERT* promoter mutations.^29^ In contrast, the frequency of *TERT* mutations is much lower in CCA cohorts, with less than 5% of cases exhibiting mutations in European populations and an even lower frequency observed in Asian cohorts.^16^

Although *TERT* rs10069690:T has been associated with increased risk of several malignancies, it has been linked to reduced risk in only very few malignancies, such as pancreatic cancer, which shares developmental similarities with CCA.^30^ We likewise observed an association with reduced iCCA risk in our exploratory UKB analysis. A potential explanation may lie in the central regenerative role of *TERT* in liver cells. As such, a subset of telomerase-expressing hepatocytes repopulated the liver during homeostasis and injury and prevented the formation of liver fibrosis, a major driver of liver carcinogenesis.^31^

While the *TERT* rs10069690:T allele was linked to a decreased risk of iCCA, its presence was also independently associated with poorer OS after CCA resection. This suggests that, despite its potential protective role in CCA cancerogenesis, the *TERT* rs10069690*:*T allele may contribute to disease progression following surgery.

The *TERT* rs10069690:T allele introduces an additional splice site in intron 4 of the *TERT* gene, resulting in the co-expression of both full-length, catalytically active *TERT* and an alternatively spliced isoform, INS1b.^12 13^ INS1b retains the ability to bind the telomerase RNA component but lacks reverse transcriptase activity and can competitively inhibit assembly of functional telomerase complexes.^14^ Increased INS1b expression has been associated with reduced telomerase activity and telomere-specific DNA damage responses.^14^ However, isoform expression is highly cell-type specific and leucocyte telomere length has not consistently differed by genotype,^13 14^ suggesting that systemic measurements may not reflect tissue-specific effects. Importantly, we did not assess *TERT* splice variant expression in tumour tissue or immune cell subsets in our cohort. Although we observed immune-related transcriptional signatures associated with overall *TERT* expression, we did not perform splice variant-specific immune profiling. Therefore, it remains unclear whether *TERT* rs10069690-associated splicing patterns modulate the tumour microenvironment or influence antitumour immune responses in CCA. Dedicated functional and spatially resolved studies will be required to clarify these mechanisms.

Beyond these telomere-related effects, the SNP likely engages non-canonical transcriptional roles which may exert predominant systemic effects. As such, in the setting of cancer, *TERT* modulates key regulators of cellular proliferation and survival including the transcription factor NF-κB, the proto-oncogene MYC and the cyclin-dependent kinase inhibitor p21 independently of telomere length.^32^

Higher intratumour *TERT* expression was associated with a non-significant trend towards shorter OS in an external iCCA cohort^16^ supporting a potential role in promoting aggressive tumour behaviour. Network analysis of gene sets co-expressed with *TERT* highlighted a significant upregulation of pathways involved in cell cycle regulation such as nuclear division, DNA repair while pathways of cell differentiation were downregulated, all of which can contribute to tumour growth and progression. These findings are in line with previous observations that across cancer entities, telomerase enzymatic activity strongly correlates with cancer stemness.^33^ We furthermore observed a downregulation of genes associated with immune responses, particularly T cell responses, in tumours with high *TERT* expression. A higher Treg signature was noted in *TERT*-high iCCA potentially reflecting the abundance of these immunosuppressive lymphoid populations and an immunosuppressive tumour microenvironment.^34 35^ Similarly, our group has previously shown that SNPs in the interleukin (IL)-1β and IL-8 pathways independently predict prognosis in iCCA and pCCA, further emphasising the importance of inflammatory and immune pathways in driving disease progression.^36 37^ However, we acknowledge that co-expression and pathway analyses were performed from bulk RNA sequencing which lacks spatial resolution and cannot distinguish between contributions from specific cell types or microenvironmental niches. In addition, publicly available datasets did not allow robust splice-variant–level correlation analyses with immune checkpoint markers such as programmed cell death protein 1 (PD-1), programmed death-ligand 1 (PD-L1) or cytotoxic T-lymphocyte-associated protein 4 (CTLA-4), limiting mechanistic inference.

Hypothesising that the non-coding *TERT* polymorphism may impact systemic protein expression we analysed the UKB proteomics dataset. While the polymorphism has been previously only associated with systemically decreased non-albumin protein and sex hormone binding globulin levels,^38^ we found that the polymorphism was associated with changes in numerous immune pathways in healthy carriers of the T allele, both homo (T/T) and heterozygous (C/T). Our findings support the hypothesis that the role of *TERT*—both in iCCA and in the systemic context of healthy individuals carrying the rs10069690 variant—may be mediated through differential transcriptional and proteomic programmes, influencing key cellular processes such as proliferation, immune modulation and differentiation.

The *TERT* rs10069690 germline variant may offer clinical value as a biomarker for both risk stratification and prognostication following surgical resection. In high-risk populations such as patients with primary sclerosing cholangitis (PSC), *TERT* rs10069690 genotyping could, if validated, contribute to risk stratification strategies; however, PSC is inconsistently coded in the UK Biobank, while our surgical cohort was too limited by sample size to allow for etiological stratification, precluding a robust assessment of this application. Similarly, stratifying the surgical cohort by iCCA and pCCA caused a loss of significance in univariable analysis despite similar directional trends. In patients with manifest disease, the *TERT* rs10069690 T allele’s association with poorer survival suggests potential as a prognostic biomarker, possibly complementing established clinicopathological factors. Prospective validation and integration into multivariable models will be essential to determine its clinical utility.

While the present study provides insights into the association between the *TERT* rs10069690:T polymorphism and CCA, several limitations should be considered. First, the initial germline analysis followed a candidate SNP approach evaluating 26 preselected variants. After Bonferroni adjustment, the association of *TERT* rs10069690 with iCCA risk would not meet the corrected significance threshold in this testing approach and should accordingly be regarded as exploratory. While our candidate-based analysis does not retain statistical significance after strict correction for multiple testing, the relevance of the *TERT* locus is supported by a recently published genome-wide meta-analysis including 893 cases of biliary tract cancer and 458 134 European-ancestry controls from the UKB, in which the *TERT* locus reached genome-wide significance.^39^ This biliary tract cancer analysis was performed as a secondary endpoint within a Genome-Wide Association Study (GWAS) primarily powered for HCC, did not distinguish CCA subtypes and the European dataset partially overlaps with our discovery population, limiting its independence as external validation. Moreover, no significant association at the *TERT* locus was reported in the Asian ancestry, suggesting potential ancestry-specific effects of the *TERT* polymorphism. As such, our findings have not been independently validated in a separate iCCA-specific cohort and prognostic association was derived from a single surgical series. Independent validation in well-annotated cohorts will be essential to determine robustness and reproducibility.

Second, CCA is a rare malignancy in Europe, resulting in limited sample sizes. Therefore, subgroup analyses including stratification by iCCA and pCCA or assessment of proteomics in individuals who later developed iCCA were underpowered. Similarly, this restricted the possibility of examining the *TERT* rs10069690 polymorphism as a biomarker in at-risk subcohorts such as in patients with a diagnosis of PSC. Our data on CCA resection are derived from a retrospectively analysed patient cohort which carries inherent limitations such as missing data, as reflected in the smaller subcohort available for multivariable analysis.

Third, although the *TERT* rs10069690:T allele was independently associated with OS, the biological explanation for a protective association in the general population but adverse prognostic implications in manifest disease remains incompletely understood. These prognostic findings should therefore also be interpreted as exploratory pending external validation. Our observational design does not establish causality between the *TERT* polymorphism and clinical outcomes, and the observed associations require validation in independent cohorts.

In summary, our findings suggest that the *TERT* rs10069690:T allele is associated with a reduced risk of developing iCCA but is also linked to poor OS in patients with manifest disease, potentially reflecting aggressive tumour phenotypes and an immune-evasive tumour microenvironment. These results are exploratory, do not survive strict correction for multiple comparisons and require independent validation in additional cohorts before clinical interpretation. The *TERT* rs10069690 polymorphism may serve as a candidate biomarker for risk stratification and prognosis in CCA, although its clinical application will require validation in independent cohorts.

## Supplementary material

10.1136/egastro-2025-100320 online supplemental file 1

## Data Availability

Data are available in a public, open access repository. Data are available upon reasonable request.

## References

[1] Khan SA, Tavolari S, Brandi G (2019). Cholangiocarcinoma: Epidemiology and risk factors. *Liver Int*.

[2] Villard C, Jorns C, Bergquist A (2024). Treatment of cholangiocarcinoma in patients with primary sclerosing cholangitis: a comprehensive review. *eGastroenterology*.

[3] Rimassa L, Khan S, Groot Koerkamp B (2025). Mapping the landscape of biliary tract cancer in Europe: challenges and controversies. *Lancet Reg Health Eur*.

[4] TLo C (2023). Outcomes of elective liver surgery worldwide: a global, prospective, multicenter, cross-sectional study. Int J Surg.

[5] Mueller M, Breuer E, Mizuno T (2021). Perihilar Cholangiocarcinoma - Novel Benchmark Values for Surgical and Oncological Outcomes From 24 Expert Centers. Ann Surg.

[6] Alvaro D, Gores GJ, Walicki J (2023). EASL-ILCA Clinical Practice Guidelines on the management of intrahepatic cholangiocarcinoma. J Hepatol.

[7] Macias RIR, Cardinale V, Kendall TJ (2022). Clinical relevance of biomarkers in cholangiocarcinoma: critical revision and future directions. Gut.

[8] Speliotes EK, Schneider CV (2025). PNPLA3 I148M Interacts With Environmental Triggers to Cause Human Disease. *Liver Int*.

[9] Miller H, Czigany Z, Lurje I (2020). Impact of Angiogenesis- and Hypoxia-Associated Polymorphisms on Tumor Recurrence in Patients with Hepatocellular Carcinoma Undergoing Surgical Resection. Cancers (Basel).

[10] Pavicevic S, Reichelt S, Uluk D (2022). Prognostic and Predictive Molecular Markers in Cholangiocarcinoma. Cancers (Basel).

[11] Liu M, Zhang Y, Jian Y (2024). The regulations of telomerase reverse transcriptase (TERT) in cancer. *Cell Death Dis*.

[12] Florez-Vargas O, Ho M, Hogshead MH (2025). Genetic regulation of TERT splicing affects cancer risk by altering cellular longevity and replicative potential. Nat Commun.

[13] Bojesen SE, Pooley KA, Johnatty SE (2013). Multiple independent variants at the TERT locus are associated with telomere length and risks of breast and ovarian cancer. Nat Genet.

[14] Killedar A, Stutz MD, Sobinoff AP (2015). A Common Cancer Risk-Associated Allele in the hTERT Locus Encodes a Dominant Negative Inhibitor of Telomerase. PLoS Genet.

[15] Lurje I, Uluk D, Pavicevic S (2023). Body composition is associated with disease aetiology and prognosis in patients undergoing resection of intrahepatic cholangiocarcinoma. Cancer Med.

[16] Dong L, Lu D, Chen R (2022). Proteogenomic characterization identifies clinically relevant subgroups of intrahepatic cholangiocarcinoma. Cancer Cell.

[17] Xu S, Hu E, Cai Y (2024). Using clusterProfiler to characterize multiomics data. Nat Protoc.

[18] Merico D, Isserlin R, Stueker O (2010). Enrichment map: a network-based method for gene-set enrichment visualization and interpretation. PLoS One.

[19] Kucera M, Isserlin R, Arkhangorodsky A (2016). AutoAnnotate: A Cytoscape app for summarizing networks with semantic annotations [version 1; peer review: 2 approved]. F1000Res.

[20] Thiele C, Hirschfeld G (2021). Cutpointr: improved estimation and validation of optimal cutpoints in r. J Stat Softw.

[21] Xie Z, Bailey A, Kuleshov MV (2021). Gene Set Knowledge Discovery with Enrichr. Curr Protoc.

[22] Shay JW, Wright WE (2019). Telomeres and telomerase: three decades of progress. Nat Rev Genet.

[23] Hasegawa K, Zhao Y, Garbuzov A (2024). Clonal inactivation of TERT impairs stem cell competition. Nature.

[24] Shay JW (2016). Role of Telomeres and Telomerase in Aging and Cancer. Cancer Discov.

[25] Shim HS, Horner JW, Wu C-J (2021). Telomerase Reverse Transcriptase Preserves Neuron Survival and Cognition in Alzheimer’s Disease Models. Nat Aging.

[26] Koh CM, Khattar E, Leow SC (2015). Telomerase regulates MYC-driven oncogenesis independent of its reverse transcriptase activity. J Clin Invest.

[27] Yuan X, Dai M, Xu D (2020). TERT promoter mutations and GABP transcription factors in carcinogenesis: More foes than friends. Cancer Lett.

[28] Gupta S, Vanderbilt CM, Lin Y-T (2021). A Pan-Cancer Study of Somatic TERT Promoter Mutations and Amplification in 30,773 Tumors Profiled by Clinical Genomic Sequencing. J Mol Diagn.

[29] Nault JC, Zucman-Rossi J (2016). TERT promoter mutations in primary liver tumors. Clin Res Hepatol Gastroenterol.

[30] He G, Song T, Zhang Y (2019). TERT rs10069690 polymorphism and cancers risk: A meta-analysis. Mol Genet Genomic Med.

[31] Lin S, Nascimento EM, Gajera CR (2018). Distributed hepatocytes expressing telomerase repopulate the liver in homeostasis and injury. Nature.

[32] Amin A, Morello M, Petrara MR (2023). Short-Term TERT Inhibition Impairs Cellular Proliferation via a Telomere Length-Independent Mechanism and Can Be Exploited as a Potential Anticancer Approach. Cancers (Basel).

[33] Noureen N, Wu S, Lv Y (2021). Integrated analysis of telomerase enzymatic activity unravels an association with cancer stemness and proliferation. Nat Commun.

[34] Werner W, Kuzminskaya M, Lurje I (2024). Overcoming Resistance to Immune Checkpoint Blockade in Liver Cancer with Combination Therapy: Stronger Together?. *Semin Liver Dis*.

[35] Wu G, Yang T, Gholami S (2025). Regulatory T cells in liver metastases: emerging and divergent roles in tumour progression. *eGastroenterology*.

[36] Lurje I, Czigany Z, Bednarsch J (2022). Genetic Variant of CXCR1 (rs2234671) Associates with Clinical Outcome in Perihilar Cholangiocarcinoma. Liver Cancer.

[37] Lurje I, Gaisa NT, Dahl E (2023). Genetic polymorphisms in interleukin-1β (rs1143634) and interleukin-8 (rs4073) are associated with survival after resection of intrahepatic cholangiocarcinoma. Sci Rep.

[38] Sinnott-Armstrong N, Tanigawa Y, Amar D (2021). Genetics of 35 blood and urine biomarkers in the UK Biobank. Nat Genet.

[39] Ghouse J, Gellert-Kristensen H, O’Rourke CJ (2025). Genome-wide meta-analysis identifies nine loci associated with higher risk of hepatocellular carcinoma development. *JHEP Rep*.

