# Supplementary material for: TERT rs10069690 variant is linked to reduced cholangiocarcinoma incidence but adverse prognosis in patients undergoing resection
Source: eGastroenterology. 2026 May 29;4(2):e100320. doi: 10.1136/egastro-2025-100320 (PMC13223632; doi:10.1136/egastro-2025-100320)
Supplement: online supplemental file 1 [file egastro-4-2-s001.pdf]

Supplementary material

Table of contents

- Supplementary Figures
  - Supplementary Figure 1
  - Supplementary Figure 2
- Supplementary Tables
  - Supplementary Table 1
  - Supplementary Table 2
  - Supplementary Table 3
  - Supplementary Table 4
  - Supplementary Table 5
  - Supplementary Table 6
  - Supplementary Table 7
  - Supplementary Table 8
  - Supplementary Table 9
  - Supplementary Table 10
  - Supplementary Table 11
  - Supplementary Table 12
  - Supplementary Table 13

Supplementary Figures

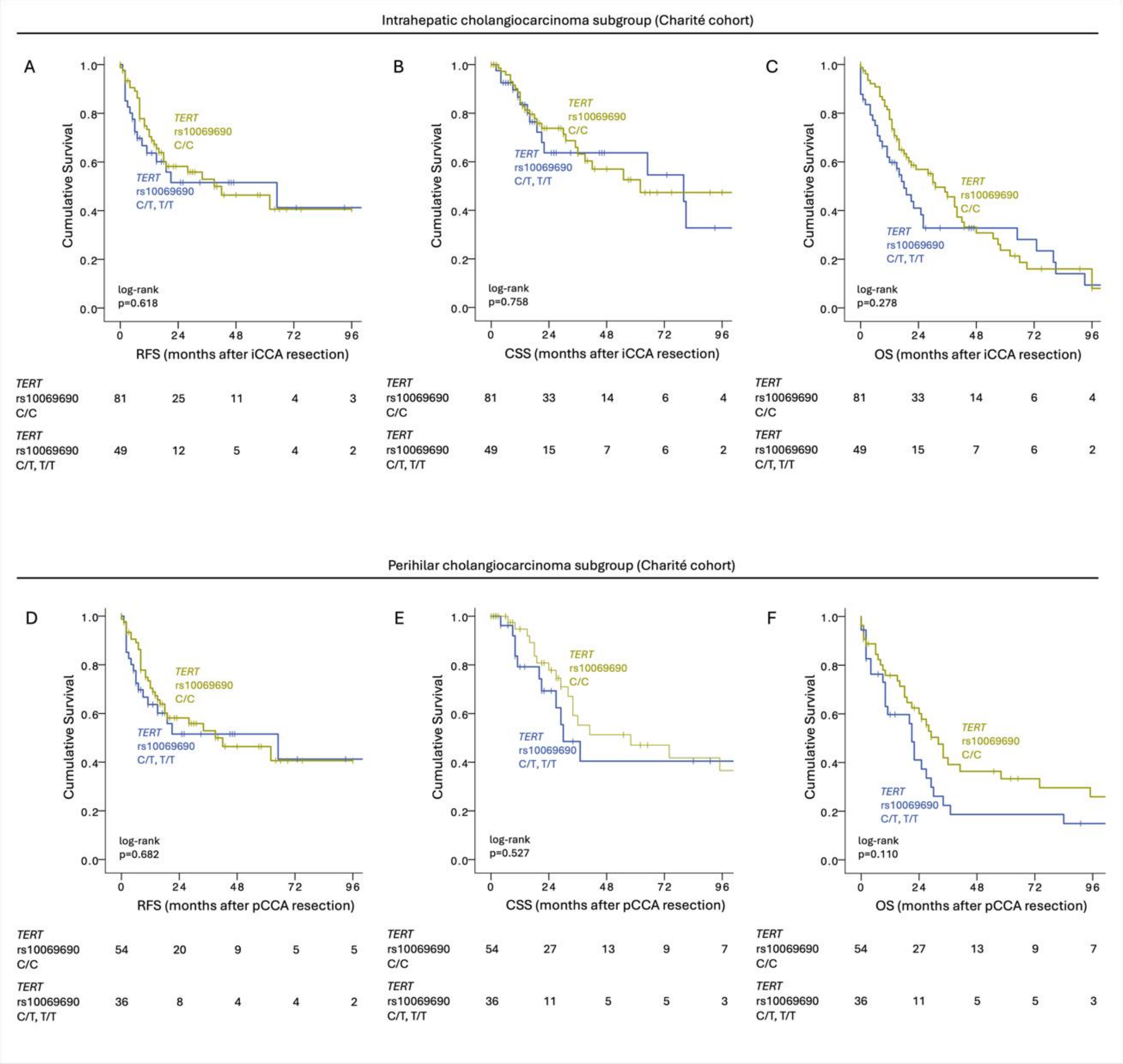

Supplementary Figure 1: Postoperative survival after iCCA and pCCA resection by *TERT* genotype
A) Recurrence-free survival (RFS), B) Cancer-specific survival (CSS), C) Overall survival (OS) after intrahepatic Cholangiocarcinoma (iCCA) resection. D) Recurrence-free survival (RFS), E) Cancer-specific survival (CSS), F) Overall survival (OS) after perihilar Cholangiocarcinoma (pCCA) resection.

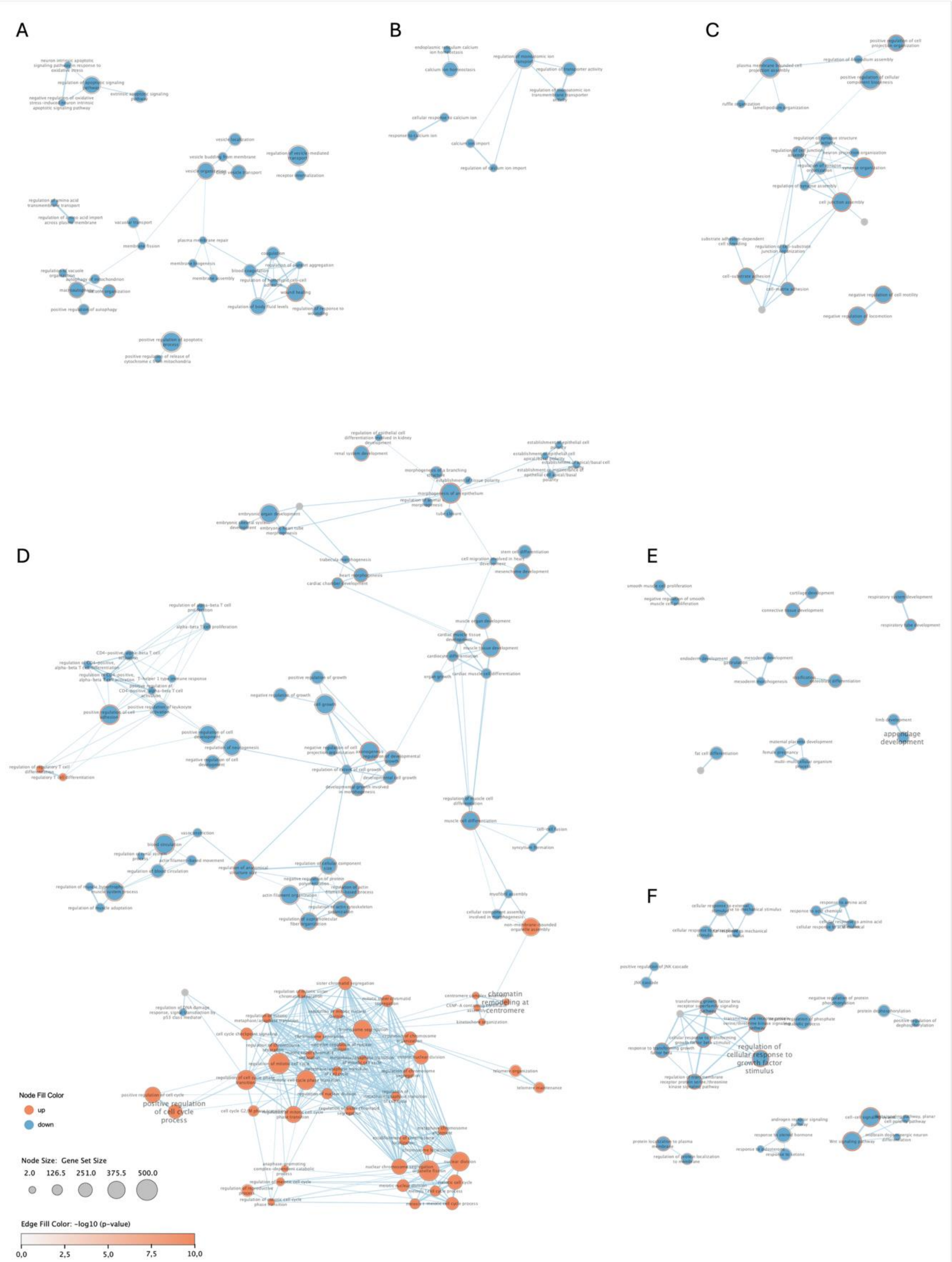

Supplementary Figure 2: Enrichment map of selected gene enrichment sets of TERT-high iCCA.

Individual nodes/gene sets are annotated with the gene ontology (GO) terminology. Depicted clusters are A) associated with vesicular transformation and apoptosis (part of the larger “signaling” cluster), B) Ca^{2+} transport and homeostasis (part of the larger “signaling” cluster), C) cell adhesion and motility, D) cell cycle and growth regulation (contains the clusters “tissue ontogeny and differentiation”, “cell cycle and growth

regulation”, “Immune regulation”), E) tissue ontogeny and differentiation, F) response to stimuli.

Gene	rsID	Risk Allele	TaqMan Assay
PNPLA3	rs738409	G	C_7241_10
TERT	rs10069690	T	C_30322061_10
HLA	rs2856718	T	C_27015374_30
MARC1	rs2642438	A	C_1235772_10
TM6SF2	rs58542926	T	C_89463510_10

Supplementary Table 1: Assays used for genotyping in the Charité cohort

Genotype	**Total, n (%)*** (n=221)	**iCCA, n (%)** (n=130)	**pCCA, n (%)** (n=91)
TERT rs10069690			
CC	135 (61.4)	81 (62.3)	54 (60.0)
CT	68 (30.9)	40 (30.8)	28 (31.1)
TT	17 (7.7)	9 (6.9)	8 (8.9)
HLA rs2856718			
CC	91 (41.6)	47 (36.4)	44 (48.9)
CT	104 (47.5)	67 (51.5)	37 (41.1)
TT	24 (11.0)	15 (11.6)	9 (10.0)
TM6SF2 rs58542926			
CC	166 (76.5)	96 (75.0)	70 (78.7)
CT	43 (19.8)	30 (23.4)	13 (14.6)
TT	8 (3.7)	2 (1.6)	6 (6.7)
PNPLA3 rs738409			
CC	125 (56.6)	72 (55.4)	53 (58.2)
CG	78 (35.3)	47 (36.2)	31 (34.1)
GG	18 (8.1)	11 (8.5)	7 (7.7)
MTARC1 rs2642438			
AA	13 (6.0)	6 (4.8)	7 (7.8)
AG	89 (41.4)	52 (41.6)	37 (41.1)
GG	113 (52.6)	67 (53.6)	46 (51.1)

Supplementary Table 2: Polymorphism genotypes in the Charité CCA cohort, divided by intrahepatic cholangiocarcinoma (iCCA) and perihilar cholangiocarcinoma (pCCA). *Genotyping was successful in n=220 (99.5%) for TERT, n=219 (99.1%) for HLA, n=217 (98.2%) for TM6SF2, n=221 (100.0%) for PNPLA3, and n=215 (97.3%) MTARC. Percentages are calculated based on the total number of successful genotypizations.

	iCCA (n=130)			pCCA (n=90#)		
	**C/C** (n=81)	**C/T, T/T** (n=49)	***p*=†**	**C/C** (n=54)	**C/T, T/T** (n=36)	***p*=†**
Age (years)	67.9 (56.7-72.4)	65.9 (61.7-73.8)	.436	64.3 (55.9-73.2)	67.0 (60.4-73.9)	.139
BMI (kg/m^2)	26.0 (22.7-28.8)	24.6 (23.0-28.3)	.583	25.5 (22.8-28.4)	24.8 (22.3-28.8)	.630
Sex, female, n (%)	35 (43.2)	19 (38.8)	.714	20 (37.0)	12 (33.3)	.823
LiMAx (µg/kg/h)	424 (320.0-506.0)	414 (336.8-504.0)	.914	371 (311.0-459.0)	347 (294.0-411.0)	.338
CA19-9 (kU/l)	23 (9.0-89.0)	75.8 (15.2-672.8)	**.012**	69 (26.2-255.5)	78.8 (33.4-1413.9)	.468
Tumor diameter (mm)	60 (40.0-90.0)	46 (35.0-70.0)	.173	42 (30.0-72.0)	57.5 (35.0-90.0)	.219
Lymph node positivity n, (%)	26 (32.1)	15 (30.6)	.834	30 (55.6)	16 (44.4)	.508
Lymphovascular invasion n, (%)	19 (23.5)	13 (26.5)	>.999	18 (33.3)	13 (36.1)	>.999
Vascular invasion, n (%)	15 (18.5)	6 (12.2)	.335	11 (20.4)	11 (30.6)	.452
Perineural invasion, n (%)	18 (22.2)	12 (24.5)	>.999	32 (59.3)	19 (52.8)	.531
R1-Resection n, (%)	23 (28.4)	13 (26.5)	.842	14 (25.9)	8 (22.2)	.804
UICC >II, n (%)	28 (34.6)	19 (38.8)	.705	30 (55.6)	19 (52.8)	.829

Supplementary Table 3: Patient characteristics in the Charité cohort by *TERT* rs10069690 genotype

Data presented as median and interquartile range if not noted otherwise.

# The cohort includes a total of n=91 patients with pCCA; however, genotyping for *TERT* rs10069690 failed in one sample.

† Mann-Whitney U Test was used for continuous variables, Chi Square Test or two-sided Fisher’s exact test for categorical variables.

Abbreviations: CA 19-9, Carbohydrate-Antigen 19-9; BMI, body mass index; iCCA, intrahepatic cholangiocellular carcinoma; pCCA perihilar cholangiocellular carcinoma; UICC, Union for International Cancer Control

**Genotype**	**Median RFS**, months (95% CI)	**Median CSS**, months (95% CI)	**Median OS**, months (95% CI)
Total cohort (N=221)			
TERT rs10069690			
CC	34 (24-45)	58 (24-92)	31 (24-38)
CT & TT	29 (0-72)	65 (1-129)	21 (17-25)
Log rank p	0.536	0.492	**0.034**
HLA rs2856718			
CC	28 (19-37)	36 (11-62)	23 (16-30)
CT & TT	28 (19-37)	74 (49-100)	25 (17-33)
Log rank p	0.469	0.402	0.962
TM6SF2 rs58542926			
CC	29 (15-43)	62 (32-92)	25 (19-32)
CT & TT	28 (4-52)	39 (0-93)	23 (13-33)
Log rank p	0.830	0.966	0.760
PNPLA3 rs738409			
CC	29 (0-70)	81 (n.a.)	25 (18-32)
CG & GG	32 (21-43)	42 (17-67)	25 (17-34)
Log rank p	0.868	0.384	0.676
MTARC1 rs2642438			
AA	19 (n.a.)	n.a.	55 (20-90)
AG & GG	29 (19-39)	58 (25-91)	24 (20-29)
Log rank p	0.696	0.139	0.051
iCCA (n=130)			
TERT rs10069690			
CC	39 (9-69)	62 (n.a.)	31 (21-41)
CT & TT	65 (0-137)	80 (4-156)	18 (11-25)
Log rank p	0.618	0.758	0.278
HLA rs2856718			
CC	34 (10-58)	42 (16-68)	26 (14-38)
CT & TT	62 (0-125)	80 (60-100)	21 (13-29)
Log rank p	0.865	0.463	0.504
TM6SF2 rs58542926			
CC	42 (6-78)	62 (27-97)	21 (13-30)

CT & TT	39 (21-57)	n.a.	36 (18-54)
Log rank p	0.566	0.160	0.108
PNPLA3 rs738409			
CC	42 (23-61)	81 (n.a.)	21 (12-30)
CG & GG	39 (0-92)	62 (29-95)	30 (14-47)
Log rank p	0.936	0.270	0.103
MTARC1 rs2642438			
AA	19 (3-35)	55 (n.a.)	48 (16-80)
AG & GG	39 (0-78)	65 (20-110)	22 (13-31)
Log rank p	0.967	0.432	0.485
pCCA (n=91)			
TERT rs10069690			
CC	29 (0-58)	58 (5-111)	32 (24-40)
CT & TT	27 (22-32)	30 (17-43)	21 (19-23)
Log rank p	0.682	0.527	0.110
HLA rs2856718			
CC	32 (17-47)	32 (22-42)	28 (19-38)
CT & TT	27 (7-47)	58 (13-103)	25 (18-32)
Log rank p	0.433	0.661	0.763
TM6SF2 rs58542926			
CC	29 (20-38)	74 (2-146)	29 (21-37)
CT & TT	16 (11-21)	24 (16-32)	18 (14-22)
Log rank p	0.361	**0.018**	**0.022**
PNPLA3 rs738409			
CC	27 (19-34)	n.a.	21 (14-28)
CG & GG	28 (14-42)	41 (12-70)	34 (25-43)
Log rank p	0.868	0.923	0.350
MTARC1 rs2642438			
AA	14 (n.a.)	n.a.	n.a.
AG & GG	27 (23-31)	37 (27-47)	37 (27-47)
Log rank p	0.552	0.199	0.122

Supplementary Table 4: Association of SNPs with postoperative survival in the total Charité cohort and separated by intrahepatic cholangiocarcinoma (iCCA) and perihilar cholangiocarcinoma (pCCA). Median recurrence-free survival (RFS), Cancer-specific

survival (CCS), ans overall survival (OS) were estimated and reported with 95% confidence intervals (CI). n.a. – estimates not reached

	**Recurrence-free survival**		**Cancer-specific survival**		**Overall survival**	
	**Median** months (95% CI)	**HR** (95% CI)	**Median** months (95% CI)	**HR** (95% CI)	**Median** months (95% CI)	**HR** (95% CI)
Sex						
Female	34 (0-83)	1 (ref)	81 (44-118)	1 (ref)	28 (5-51)	0.736 (0.479-1.132)
Male	18 (7-77)	1.206 (0.692-2.102)	62 (24-100)	1.374 (0.725-2.605)	23 (11-35)	1 (ref)
Log rank p	0.502		0.326		0.157	
Age						
≤65	19 (3-35)	1 (ref)	55 (23-87)	1 (ref)	30 (13-47)	1 (ref)
>65	n.a.	0.549 (0.316-0.954)	81 (n.a.)	0.711 (0.378-1.335)	22 (12-32)	1.065 (0.688-1.649)
Log rank p	**0.029**		0.284		0.775	
BMI, kg/m^2						
≤25	34 (n.a.)	1 (ref)	81 (26-136)	1 (ref)	17 (6-29)	1 (ref)
>25	62 (16-108)	0.831 (0.470-1.468)	62 (33-92)	1.053 (0.548-2.023)	25 (16-34)	0.862 (0.559-1.329)
Log rank p	0.517		0.877		0.497	
Tumor Stage						
UICC I & II	n.a.	1 (ref)	81 (56-106)	1 (ref)	28 (9-47)	1 (ref)
UICC III & IV	16 (9-24)	1.851 (1.048-3.269)	36 (28-45)	1.719 (0.893-3.308)	23 (13-33)	1.222 (0.784-1.906)
Log rank p	**0.029**		0.099		0.370	
Tumor Grading						
G1 & G2	62 (33-91)	1 (ref)	65 (35-95)	1 (ref)	26 (12-40)	1 (ref)
G3	18 (12-24)	1.411 (0.794-2.509)	n.a.	1.373 (0.715-2.637)	21 (10-32)	1.099 (0.692-1.747)
Log rank p	0.232		0.765		0.695	
Resection status						
R0	n.a.	1 (ref)	n.a.	1 (ref)	26 (11-41)	1 (ref)
R1/Rx	14 (7-21)	1.709 (0.961-3.048)	39 (20-58)	1.864 (0.971-3.577)	17 (3-31)	1.555 (0.992-2.437)
Log rank p	0.062		0.056		0.050	
pT category						
T1-2	42 (n.a.)	1 (ref)	81 (n.a.)	1 (ref)	25 (11-39)	1 (ref)
T3-4	19 (16-22)	1.128 (0.618-2.057)	31 (21-41)	1.789 (0.922-3.474)	18 (8-28)	1.278 (0.801-2.038)
Log rank p	0.691		0.080		0.296	

pN category						
N0	n.a.	1 (ref)	n.a.	1 (ref)	43 (11-75)	1 (ref)
N1	12 (8-16)	3.030 (1.608-5.710)	30 (10-50)	3.910 (1.810-9.446)	16 (11-21)	2.778 (1.627-4.742)
Log rank p	**<0.001**		**<0.001**		**<0.001**	
Lymphovascular invasion						
L0	n.a.	1 (ref)	n.a.	1 (ref)	39 (24-54)	1 (ref)
L1	10 (7-13)	3.720 (2.034-6.802)	19 (17-21)	4.712 (2.287-9.665)	18 (11-26)	2.249 (1.326-3.814)
Log rank p	**<0.001**		**<0.001**		**0.002**	
Vascular invasion						
V0	62 (20-104)	1 (ref)	n.a.	1 (ref)	26 (8-44)	1 (ref)
V1	19 (0-42)	1.522 (0.775-2.989)	36 (27-45)	1.894 (0.909-3.950)	30 (10-50)	1.254 (0.709-2.218)
Log rank p	0.213		0.082		0.431	
Perineural invasion						
Pn0	n.a.	1 (ref)	n.a.	1 (ref)	69 (n.a.)	1 (ref)
Pn1	19 (13-26)	1.133 (0.512-2.511)	36 (5-67)	1.603 (0.550-4.677)	26 (11-41)	1.748 (0.721-4.237)
Log rank p	0.756		0.381		0.208	

Supplementary Table 5: Patient and tumor characteristics in association with long-term outcomes in the iCCA cohort

Median recurrence-free survival (RFS), Cancer-specific survival (CCS), and overall survival (OS) were estimated and reported with 95% confidence intervals (CI) in the subcohoert of patients with intrahepatic cholangiocarcinoma (iCCA). n.a. – estimates/ median not reached. Hazard ratios (HR) were reported with 95% CIs.

	Recurrence-free survival		Cancer-specific survival		Overall survival	
	**Median** months (95% CI)	**HR** (95% CI)	**Median** months (95% CI)	**HR** (95% CI)	**Median** months (95% CI)	**HR** (95% CI)
Sex						
Female	37 (n.a.)	1 (ref)	n.a.	1 (ref)	22 (8-37)	1.119 (0.643-1.946)
Male	25 (9-41)	1.993 (0.940-4.222)	34 (26-42)	1.805 (0.767-4.245)	27 (19-35)	1 (ref)
Log rank p	0.089		0.167		0.687	
Age						
≤65	16 (4-28)	1 (ref)	41 (0-83)	1 (ref)	32 (19-45)	1 (ref)
>65	29 (24-34)	0.708 (0.370-1.358)	37 (1-73)	0.981 (0.471-2.042)	22 (18-26)	1.307 (0.768-2.225)
Log rank p	0.529		0.958		0.315	
BMI, kg/m^2						
≤25	14 (0-29)	1 (ref)	58 (10-106)	1 (ref)	24 (17-31)	1 (ref)
>25	29 (24-34)	0.666 (0.352-1.261)	36 (29-43)	0.978 (0.465-2.057)	27 (19-36)	1.077 (0.634-1.831)
Log rank p	0.295		0.954		0.780	
Tumor Stage						
UICC I & II	31 (7-57)	1 (ref)	n.a.	1 (ref)	34 (22-46)	1 (ref)
UICC III & IV	16 (4-28)	1.850 (0.949-3.603)	34 (21-47)	2.012 (0.052-4.253)	21 (15-27)	2.261 (1.299-3.936)
Log rank p	0.067		0.061		**0.003**	
Tumor Grading						
G1 & G2	29 (23-36)	1 (ref)	58 (13-104)	1 (ref)	28 (20-36)	1 (ref)
G3	15 (11-19)	1.535 (0.740-3.187)	29 (18-40)	1.552 (0.655-3.673)	21 (10-32)	1.431 (0.786-2.606)
Log rank p	0.245		0.311		0.232	
Resection status						
R0	29 (24-34)	1 (ref)	n.a.	1 (ref)	28 (23-34)	1 (ref)
R1/Rx	24 (10-40)	1.617 (0.799-3.271)	34 (20-48)	1.746 (0.793-3.844)	19 (4-34)	1.742 (0.991-3.060)
Log rank p	0.176		0.159		**0.048**	
pT category						
T1-2	29 (0-100)	1 (ref)	n.a.	1 (ref)	41 (0-114)	1 (ref)
T3-4	24 (11-39)	1.958 (0.939-4.084)	36 (28-44)	2.564 (1.085-6.059)	21 (14-28)	2.280 (1.216-4.276)
Log rank p	0.068		**0.025**		**0.008**	

pN category						
N0	29 (23-35)	1 (ref)	n.a.	1 (ref)	58 (9-107)	1 (ref)
N1	25 (12-38)	1.626 (0.829-3.189)	34 (19-49)	2.911 (1.323-6.402)	18 (9-27)	3.129 (1.740-5.627)
Log rank p	0.153		**0.005**		**<0.001**	
Lymphovascular invasion						
L0	29 (20-39)	1 (ref)	74 (5-143)	1 (ref)	32 (26-38)	1 (ref)
L1	25 (13-37)	1.383 (0.684-2.798)	34 (0-121)	1.649 (0.727-3.738)	19 (11-27)	1.637 (0.932-2.875)
Log rank p	0.364		0.225		0.080	
Vascular invasion						
V0	32 (21-42)	1 (ref)	74 (0-152)	1 (ref)	29 (22-36)	1 (ref)
V1	11 (6-15)	4.224 (1.926-9.263)	27 (13-41)	3.330 (1.401-7.910)	19 (4-34)	1.951 (1.040-3.658)
Log rank p	**<0.001**		**0.004**		**0.032**	
Perineural invasion						
Pn0	49 (2-96)	1 (ref)	n.a.	1 (ref)	29 (16-42)	1 (ref)
Pn1	15 (14-16)	2.419 (1.231-4.757)	32 (27-37)	2.383 (1.096-5.179)	21 (16-26)	1.745 (1.016-2.995)
Log rank p	**0.008**		**0.023**		**0.039**	

Supplementary Table 6: Patient and tumor characteristics in association with long-term outcomes in the pCCA cohort

Median recurrence-free survival (RFS), Cancer-specific survival (CCS), and overall survival (OS) were estimated and reported with 95% confidence intervals (CI) in the subcohort of patients with perihilar cholangiocarcinoma (pCCA). n.a. – estimates/ median not reached. Hazard ratios (HR) were reported with 95% CIs.

	n*	Hazard ratio (95%CI)	*p*
TERT rs10069690			
C/C	101	1 (reference)	**0.001**
C/T & T/T	62	2.088 (1.354-3.204)	
**pN category**			
N0	90	1 (reference)	**0.003**
N1	73	2.894 (1.419-5.903)	
**Resection status**			
R0	121	1 (reference)	**0.016**
R1/ Rx	42	1.764 (1.109-2.804)	
**Vascular invasion**			
V0	125	1 (reference)	.235
V1	38	1.375 (0.813-2.325)	
**Tumor stage**			
UICC I & II	85	1 (reference)	0.407
UICC III & IV	78	1.315 (0.689-2.510)	
**Lymphovascular invasion**			
L0	106	1 (reference)	0.852
L1	57	1.048 (0.639-1.720)	

Supplementary Table 7: Multivariable analysis of overall survival in the Charité Berlin cohort

* 163 patients with complete data were included in the model

	n*	Hazard ratio (95%CI)	*p*
TERT **rs10069690**			
C/C	57	1 (reference)	**0.009**
C/T & T/T	33	2.219 (1.223-4.024)	
**pN category**			
N0	55	1 (reference)	0.186
N1	35	1.866 (0.740-4.704)	
**Resection status**			
R0	67	1 (reference)	**0.044**
R1/ Rx	23	1.930 (1.017-3.663)	
**Vascular invasion**			
V0	72	1 (reference)	.750
V1	18	1.128 (0.537-2.368)	
**Tumor stage**			
UICC I & II	53	1 (reference)	0.978
UICC III & IV	37	1.010 (0.494-2.065)	
**Lymphovascular invasion**			
L0	61	1 (reference)	0.440
L1	29	1.376 (0.612-3.094)	

Supplementary Table 8: Multivariable analysis of overall survival in iCCA, including *TERT* rs10069690 genotype

	n*	Hazard ratio (95%CI)	*p*
TERT rs10069690			
C/C	44	1 (reference)	**0.048**
C/T & T/T	29	1.954 (1.006-3.794)	
**pN category**			
N0	35	1 (reference)	0.873
N1	38	25014 (0.0-2.519E+58)	
**Resection status**			
R0	54	1 (reference)	0.152
R1/ Rx	19	1.654 (0.831-3.294)	
**Vascular invasion**			
V0	53	1 (reference)	0.474
V1	20	1.357 (0.589-3.127)	
**Tumor stage**			
UICC I & II	32	1 (reference)	0.886
UICC III & IV	41	0.000 (0.000-1.11E+50)	
**Lymphovascular invasion**			
L0	45	1 (reference)	0.927
L1	28	0.969 (0.491-1.912)	

Supplementary Table 9: Multivariable analysis of overall survival in pCCA, including *TERT* rs10069690 genotype

	n*	Hazard ratio (95%CI)	*p*
**TM6SF2** rs58542926			
C/C	58	1 (reference)	**0.016**
C/T & T/T	14	2.462 (1.182-5.129)	
**pN category**			
N0	35	1 (reference)	0.913
N1	38	42473 (0.0-1.939E+87)	
**Resection status**			
R0	54	1 (reference)	0.130
R1/ Rx	19	1.678 (0.859-3.278)	
**Vascular invasion**			
V0	53	1 (reference)	0.166
V1	20	1.763 (0.790-3.930)	
**Tumor stage**			
UICC I & II	32	1 (reference)	0.919
UICC III & IV	41	0.000 (0.000-2.5E+78)	
**Lymphovascular invasion**			
L0	45	1 (reference)	0.845
L1	28	0.931 (0.457-1.899)	

Supplementary Table 10: Multivariable analysis of overall survival in pCCA, including TM6SF2 genotype

	n	Hazard ratio (95%CI)	*p*
**TNM stage**			
IA	47	1 (reference)	
IB	31	0.537 (0.056-5.165)	**0.590**
II	76	7.013 (2.129-23.097)	**0.001**
IIIA	5	18.971 (4.488-80.191)	**6.295E-05**
IIIB	72	11.048 (3.363-36.294)	**7.544E-05**
IV	13	24.060 (6.452-89.729)	**2.179E-06**
**Age** (continuous)			
mean	60.6 years	1.031 (1.008-1.056)	**0.007**
TERT expression			
low	68	1 (reference)	0.110
high	176	1.612 (0.898-2.892)	
**Sex**			
female	106	1 (reference)	0.655
male	138	1.110 (0.702-1.755)	

Supplementary Table 11: Multivariable analysis of factors associated with overall survival (OS) in the iCCA cohort by Dong et al [1].

Term	p-value	q-value	Overlap genes
**Top 10 upregulated significant GO terms in Heterocygous (C/T) individuals in UK Biobank (n=19,848)**			
Lymphocyte Differentiation (GO:0030098)	2.778450e-07	0.000076	*CR2, FLT3, AXL, KIT*
Regulation Of Cell Population Proliferation (GO:0042127)	4.216812e-06	0.000573	*CCL14, TNXB, ERBB3, FLT3, KIT, CXCL1*
B Cell Differentiation (GO:0030183)	1.049172e-05	0.000746	*CR2, FLT3, KIT*
Positive Regulation Of Phosphatidylinositol 3-Kinase Signaling (GO:0014068)	1.096451e-05	0.000746	*ERBB3, FLT3, KIT]*
Cytokine-Mediated Signaling Pathway (GO:0019221)	1.738340e-05	0.000787	*CCL14, FLT3, KIT, CXCL1*
Regulation Of Kinase Activity (GO:0043549)	2.054594e-05	0.000787	*ERBB3, FLT3, AXL*
Regulation Of Phosphatidylinositol 3-Kinase Signaling (GO:0014066)	2.439214e-05	0.000787	*ERBB3, FLT3, KIT*
Transmembrane Receptor Protein Tyrosine Kinase Signaling Pathway (GO:0007169)	2.572746e-05	0.000787	*ERBB3, FLT3, AXL, KIT*
B Cell Activation (GO:0042113)	2.605437e-05	0.000787	*CR2, FLT3, KIT*
Hemopoiesis (GO:0030097)	3.445866e-05	0.000787	*NOTCH2, FLT3, KIT*
Top 10 downregulated significant GO terms in Heterocygous (C/T) individuals in UK Biobank (n=19,848)			
Negative Regulation Of Mast Cell Activation (GO:0033004)	0.003745	0.05028	*[CD300LF]*

Term	p-value	q-value	Overlap genes
Positive Regulation Of Apoptotic Cell Clearance (GO:2000427)	0.005239	0.05028	*[CD300LF]*
Regulation Of Apoptotic Cell Clearance (GO:2000425)	0.005239	0.05028	*[CD300LF]*
Regulation Of Natural Killer Cell Mediated Cytotoxicity Directed Against Tumor Cell Target (GO:0002858)	0.005239	0.05028	*[CEACAM1]*
Skeletal System Development (GO:0001501)	0.005432	0.05028	*[BGLAP, MATN3]*
Positive Regulation Of Actin Cytoskeleton Reorganization (GO:2000251)	0.005985	0.05028	*[CCL27]*
Regulation Of Endothelial Cell Differentiation (GO:0045601)	0.005985	0.05028	*[CEACAM1]*
Negative Regulation Of Leukocyte Degranulation (GO:0043301)	0.005985	0.05028	*[CEACAM1]*
Response To Vitamin (GO:0033273)	0.005985	0.05028	*[BGLAP]*
Response To Vitamin D (GO:0033280)	0.005985	0.05028	*[BGLAP]*

Supplementary Table 12: Top 10 upregulated significant GO terms in heterocygous (C/T) individuals (n=19.848) compared to homozygous individuals (C/C, n=27.520) in proteomics of UK Biobank controls.

Term	p-value	q-value	Overlap genes
Top 10 upregulated significant GO terms in Homocygous (T/T) individuals in UK Biobank (n=3895)			
Cytokine-Mediated Signaling Pathway (GO:0019221)	2.935283e-09	0.000003	*[CCL14, CSF1R, FLT3, CSF2RB, LILRB1, CXCL1, CXCL5, IL17RA, CCL7, KIT, CCL3, IL7R, CCL15]*
Positive Regulation Of Cell Population Proliferation (GO:0008284)	1.742771e-08	0.000009	*[CD86, CCL14, CSF1R, TNXB, MEGF10, FLT3, NTRK3, FLT4, CSF2RB, EGFR, CXCL5, TCL1A, ERBB4, CCN4, CTSH, IL7R]*
Cellular Response To Cytokine Stimulus (GO:0071345)	2.549277e-08	0.000009	*[CCL14, CSF1R, ASAH2, FLT3, CSF2RB, LILRB1, TCL1A, CCL7, AXL, KIT, CCL3, IL7R, CCL15]*
Positive Regulation Of ERK1 And ERK2 Cascade (GO:0070374)	8.490789e-08	0.000022	*[NOTCH2, CCL14, CSF1R, CCL7, ERBB4, NTRK3, FLT4, CCL3, EGFR, CCL15]*
Neutrophil Chemotaxis (GO:0030593)	1.536290e-07	0.000028	*[CCL14, CCL7, ITGB2, CCL3, CXCL1, CXCL5, CCL15]*
Regulation Of ERK1 And ERK2 Cascade (GO:0070372)	1.658065e-07	0.000028	*[NOTCH2, CCL14, CSF1R, CCL7, ERBB4, NTRK3, FLT4, CCL3, EZR, EGFR, CCL15]*
Granulocyte Chemotaxis (GO:0071621)	2.058694e-07	0.000029	*[CCL14, CCL7, ITGB2, CCL3, CXCL1, CXCL5, CCL15]*
Positive Regulation Of MAPK Cascade (GO:0043410)	2.308006e-07	0.000029	*[NOTCH2, CCL14, CSF1R, CCL7, ERBB4, FLT3, NTRK3, FLT4, KIT, CCL3, EGFR, CCL15]*
Neutrophil Migration (GO:1990266)	2.981596e-07	0.000034	*[CCL14, CCL7, ITGB2, CCL3, CXCL1, CXCL5, CCL15]*
Regulation Of Cell Population Proliferation (GO:0042127)	3.749924e-07	0.000038	*[CD86, CCL14, CSF1R, TNXB, TFRC, FLT3, NTRK3, FLT4, CXCL1, SOD2, EGFR, CXCL5, TCL1A, ERBB4, KIT, CTSH, IL7R, CD33]*
Top 10 downregulated significant GO terms in Homocygous (T/T) individuals in UK Biobank (n=3895)			
Cytokine-Mediated Signaling Pathway (GO:0019221)	1.181329e-10	2.122849e-07	*[YAP1, CCL24, IL15RA, CCL23, CXCL9, CCL11, IL31RA, OSM, LILRA2, LILRB4, CSF2RA, TNFRSF1A, CEACAM1, CXCL12, IL2RA, IL12B, XCL1, CCL2, CD300LF, IL6R]*

Inflammatory Response (GO:0006954)	1.426666e-09	1.281859e-06	*[AOC3, GGT5, IL22, CCL24, CCL23, CXCL9, CCL11, VCAM1, IL10RB, ADM, AGER, TNFRSF1A, CRHBP, IL2RA, CRH, XCL1, CCL2, TNFRSF4]*
Positive Regulation Of MAPK Cascade (GO:0043410)	3.155939e-09	1.890407e-06	*[CCL24, CD74, EDN1, CCL23, CCL11, GDF15, OSM, CXCL17, TGFA, TREM2, FGF2, AGER, NELL1, ADAM9, XCL1, CCL2, GHRL, CD36, FGFR4, TDGF1]*
Positive Regulation Of Leukocyte Chemotaxis (GO:0002690)	2.410013e-08	1.082698e-05	*[CXCL12, MDK, RARRES2, SERPINE1, CXCL17, XCL1, IL6R, PLA2G7, PGF]*
Regulation Of ERK1 And ERK2 Cascade (GO:0070372)	9.923045e-08	3.114710e-05	*[CCL24, CD74, CCL23, CCL11, ADIPOQ, CXCL17, TREM2, FGF2, AGER, NELL1, CEACAM1, TIMP3, XCL1, CCL2, CD36, FGFR4]*
Positive Regulation Of Peptidyl-Tyrosine Phosphorylation (GO:0050731)	1.039970e-07	3.114710e-05	*[EFNA1, CD74, NELL1, HGF, CLEC6A, IL31RA, OSM, IL12B, TREM2, IL6R, TDGF1, TNFRSF1A]*
Regulation Of Peptidyl-Tyrosine Phosphorylation (GO:0050730)	2.145887e-07	5.508799e-05	*[EFNA1, CD74, NELL1, HGF, CLEC6A, OSM, IL12B, TREM2, IL6R, TDGF1]*
Positive Regulation Of Epithelial Cell Proliferation (GO:0050679)	4.915013e-07	1.009624e-04	[YAP1, CCL24, NELL1, EGFL7, CDH3, CCL11, MDK, TNFSF12, TGFA, FGF2, PGF]
Positive Regulation Of ERK1 And ERK2 Cascade (GO:0070374)	5.056546e-07	1.009624e-04	[CCL24, CD74, CCL23, CCL11, CXCL17, TREM2, FGF2, AGER, NELL1, XCL1, CCL2, CD36, FGFR4]
Positive Regulation Of Chemotaxis (GO:0050921)	1.259008e-06	2.262437e-04	NELL1, MDK, RARRES2, XCL1, TREM2, FGF2, IL6R

Supplementary Table 13: Top 10 upregulated significant GO terms in homocygous (T/T) individuals (n=3.895) compared to homozygous individuals (C/C, n=27.520) in proteomics of UK Biobank controls.

1 Dong L, Lu D, Chen R, Lin Y, Zhu H, Zhang Z*, et al.* Proteogenomic characterization identifies clinically relevant subgroups of intrahepatic cholangiocarcinoma. Cancer cell 2022;**40**:70-87.e15.
