# Supplementary material for: TERT rs10069690 variant is linked to reduced cholangiocarcinoma incidence but adverse prognosis in patients undergoing resection
Source: eGastroenterology. 2026 May 29;4(2):e100320. doi: 10.1136/egastro-2025-100320 (PMC13223632; doi:10.1136/egastro-2025-100320)
Supplement: Supplementary file 2 [file egastro-2025-100320.reviewer_comments.pdf]

PEER REVIEW HISTORY

eGastroenterology publishes all reviews undertaken for accepted manuscripts. Reviewers are asked to complete a checklist review form and are provided with free text boxes to elaborate on their assessment. These free text comments are reproduced below.

ARTICLE DETAILS

**TITLE (PROVISIONAL)**	The TERT rs10069690 variant is linked to reduced cholangiocarcinoma incidence, but adverse prognosis in patients undergoing resection
**AUTHORS**	Lurje, Isabella; Pein, Justus; Horn, Paul; Uluk, Deniz; Kohlhepp, Marlene; Phan, Minh Duc; Niklisch, Saskia; Schliephake, Frederik; Jakhar, Niharika; Schneider, Kai Markus; Roßner, Florian; Horst, David; Pratschke, Johann; Tacke, Frank; Schneider, Carolin; Lurje, Georg

VERSION 1 - REVIEW

**REVIEWER NAME**	***Wang, Xinwei***
**REVIEWER AFFILIATION**	N/A
**REVIEWER CONFLICT OF INTEREST**	No
**AI DISCLOSURE**	No, I have not used AI
**DATE REVIEW RETURNED**	05-Nov-2025

**GENERAL COMMENTS**	This manuscript explores germline variations in relation to intrahepatic cholangiocarcinoma (iCCA) risk. It applies UK Biobank data to determine CCA risk and then assesses the top candidate allele for its association with survival of CCA patients post resection. The topic is clinically relevant. However, several major issues limit the strength of the conclusions and require substantial revision.<br><br>1) The initial SNP screen (a candidate approach) tests 23 variants without adjusting for multiple test. The reported nominal associations of one variant at rs10069690 (e.g., p = 0.008) do not meet an appropriate corrected significance threshold (~0.0022 Bonferroni). Without multiple-testing adjustment, the primary germline association should be considered exploratory. This is the major deficiency of this study. The candidate approach instead of a genome wide approach also limits the strength of this study.<br><br>2) Another key deficiency of this study is the lack of an independent validation. The iCCA risk association is derived from UK Biobank only using a candidate screening approach and is not reproduced in an independent cohort. The prognostic association is likewise limited to a single surgical cohort. The results for iCCA risk and risk for post-surgery relapse are inconsistent, which questions a biological plausibility. These findings must be validated independently.<br><br>3) As the current results are not conclusive, please revise language to reflect the exploratory (preliminary) nature of the findings (e.g., “associated with” rather than “predicts” or “drives”). Suggested language in the summary: “these results are exploratory, do not survive correction for multiple comparisons, and require independent validation in additional cohorts before interpretation.”

	4) The methodology lacks analytic details. Please provide explicit covariate sets, ancestry control methods, and QC thresholds used in UK Biobank analyses. Consider providing code or scripts to enhance transparency.<br><br>5) In the limitations section, state explicitly about the preliminary nature of the finding as the SNP association was derived from a discovery cohort that does not survive strict multiple-testing correction. Prognostic effects are problematic as they raise questions about biological plausibility. Therefore, these findings should be interpreted as preliminary and hypothesis-generating.

**REVIEWER NAME**	***Lafdil, Fouad***
**REVIEWER AFFILIATION**	INSERM, CRSA
**REVIEWER CONFLICT OF INTEREST**	No
**AI DISCLOSURE**	No, I have not used AI
**DATE REVIEW RETURNED**	23-Nov-2025

**GENERAL COMMENTS**	In this study, Lurje et al. propose to determine the potential impact of TERT rs10069690 variant on the incidence and prognosis of cholangiocarcinoma according to its phenotype. The study combined transcriptomic and proteomic approaches on a local constituted cohort of CCA patients at Charité institute, UK Biobank and on online available databases.<br><br>1. The authors discussed the potential mechanisms by which the spliced variants drive tumorigenesis, but it should be also of interest to further discuss the potential link between TERT splice variants and CCA immunity. In other words, how these splice variants could modulate the tumor microenvironment, and/or affect the tumor sensitivity to the antitumor immune response (immune evasion and/or immune suppression).<br><br>2. TERT splicing analysis with immune profiling could reveal novel therapeutic targets. Are there any results that could be explored in the used databases or on the studied cohort to identify a potential impact of TERT splice variants on immune checkpoint expressions?

VERSION 1 – AUTHOR RESPONSE

Reviewer: 1
Comments to the Author
This manuscript explores germline variations in relation to intrahepatic cholangiocarcinoma (iCCA) risk. It applies UK Biobank data to determine CCA risk and then assesses the top candidate allele for its association with survival of CCA patients post resection. The topic is clinically relevant. However, several major issues limit the strength of the conclusions and require substantial revision.

1) The initial SNP screen (a candidate approach) tests 23 variants without adjusting for multiple test. The reported nominal associations of one variant at rs10069690 (e.g., p = 0.008) do not meet an appropriate corrected significance threshold (~0.0022 Bonferroni). Without multiple-testing adjustment, the primary germline association should be considered exploratory. This is the major deficiency of this study. The candidate approach instead of a genome wide approach also limits the strength of this study.

We agree and have revised the manuscript to explicitly frame the germline association as exploratory and hypothesis-generating. Accordingly, we have made the following modifications:

1. Limitations section (Discussion)
   We now explicitly state that: The candidate-based analysis evaluated 23 variants without genome-wide correction and the rs10069690 association does not meet Bonferroni-adjusted significance; The findings must therefore be interpreted as exploratory (ll. 532-535).

2. Summary paragraph: We revised the conclusion to state explicitly: "These results are exploratory, do not survive strict correction for multiple comparisons, and require independent validation in additional cohorts before clinical interpretation." (ll. 583-585).

3. GWAS contextualization (Discussion)
   We now reference the recent genome-wide meta-analysis (893 biliary tract cancer cases, 458,134 controls of European ancestry) in which the TERT locus reached genome-wide significance (see ll. 535-549). This finding is presented as contextual support only and not as independent validation, particularly given partial dataset overlap with UK Biobank. Importantly, in contrast to our candidate-based approach, this independent genome-wide analysis in all biliary cancer subsets applied appropriate genome-wide correction thresholds, under which the TERT locus reached statistical significance.

2) Another key deficiency of this study is the lack of an independent validation. The iCCA risk association is derived from UK Biobank only using a candidate screening approach and is not reproduced in an independent cohort. The prognostic association is likewise limited to a single surgical cohort. The results for iCCA risk and risk for post-surgery relapse are inconsistent, which questions a biological plausibility. These findings must be validated independently.

We also agree that a genome-wide approach would provide stronger inferential validity; however, given the rarity of iCCA, even large population resources remain limited in case numbers. We have strengthened the manuscript accordingly. We have clarified the absence of independent validation, addressed the issue of biological plausibility, and revised the manuscript to reflect the exploratory nature of the findings.

1. Explicit statement of lack of independent validation (Discussion, p. 18-19)
   We now clearly state that:
   - The iCCA risk association has not been validated in an independent iCCA-specific cohort (ll. 535-549).
   - The prognostic association derives from a single surgical series and the associated underpowered statistics (ll. 551-553).
   - Independent validation is required (ll. 548-549, ll. 562-563).
2. Biological plausibility clarification (Discussion)
   After discussing the discordant risk vs survival findings, we acknowledge:
   - We now explicitly discuss the context-dependent role of TERT in tumor initiation versus progression, which may account for the discordant directionality of risk and survival associations (ll. 393-429)
   - That this discordance underscores the preliminary nature of the findings.
3. Exploratory framing
   Causal wording such as "a critical factor", "predicts" or "drives" was revised to cautious phrasing, e.g. "associated with" where applicable, e.g. in l. 363, ll. 486-487, ll. 509-510. We replaced wording such as "valuable biomarker" with "may serve as a candidate biomarker" (l. 586).

3) As the current results are not conclusive, please revise language to reflect the exploratory (preliminary) nature of the findings (e.g., "associated with" rather than "predicts" or "drives").

Suggested language in the summary: “these results are exploratory, do not survive correction for multiple comparisons, and require independent validation in additional cohorts before interpretation.”

Thank you for this point.

We revised language throughout the manuscript to avoid causal interpretation. We replaced causal terms (“predicts”, “drives”) with association-based phrasing, added an explicit exploratory disclaimer in the limitations section regarding validation (p. 18 ll. 546-549; ll. 559-563) and closely following the reviewer’s suggested wording in the final summary paragraph (p. 19, ll. 583-585). Please also see our response to point 2 (under paragraph number 3 of the response).

4) The methodology lacks analytic details. Please provide explicit covariate sets, ancestry control methods, and QC thresholds used in UK Biobank analyses. Consider providing code or scripts to enhance transparency.

We have expanded the Methods section to improve analytic transparency and reproducibility. Modifications were made to the methods section (p. 6-7), where we now explicitly state the covariates included in regression models, the population stratification control approach and variant quality control thresholds.

“For each SNP in UKB, we fitted logistic regression models with C221 case status as the dependent variable and genotype coded as effect-allele count (0/1/2). The prespecified covariate set was age at baseline (UKB field 21003-0.0), sex (31-0.0), body mass index (21001-0.0), and genetic principal components PC1–PC10 (22009-0.1 to 22009-0.10). Population structure was controlled by including PC1–PC10 in all models” (ll.199-204).

“Polymorphisms were selected with a sufficient minor allele frequency (MAF>=0.01) and based on their reported involvement in liver disease, particularly primary liver cancer. The candidate variants were selected from loci with established relevance to liver disease and that have been repeatedly evaluated in prior population-genetic association studies, including analyses in UK Biobank. Therefore, we used UK Biobank’s centrally processed genotypes/imputed dosages and restricted to variants with MAF ≥ 0.01. As UK Biobank centrally applies standardized QC procedures, no additional study-specific variant-level filters were imposed. Genotyping of the selected variants was conducted in a total of 475089 subjects in UKB. Candidate variants were obtained either from directly genotyped array data or from the UK Biobank imputed-v3 resource” (ll. 115-124).

5) In the limitations section, state explicitly about the preliminary nature of the finding as the SNP association was derived from a discovery cohort that does not survive strict multiple-testing correction. Prognostic effects are problematic as they raise questions about biological plausibility. Therefore, these findings should be interpreted as preliminary and hypothesis-generating.

This is now explicitly stated in the limitations (p. 18-19), we clarify that the SNP association does not survive strict multiple-testing correction (ll. 532-537), the findings are preliminary and hypothesis-generating (ll. 546-548) and that prognostic associations require independent confirmation (ll. 548-549). We also tempered the final summary to reflect this framing (p. 20, ll. 583-585).

Reviewer: 2

Comments to the Author
In this study, Lurje et al. propose to determine the potential impact of TERT rs10069690 variant on the incidence and prognosis of cholangiocarcinoma according to its phenotype. The study combined transcriptomic and proteomic approaches on a local constituted cohort of CCA patients at Charité institute, UK Biobank and on online available databases.

1. The authors discussed the potential mechanisms by which the spliced variants drive tumorigenesis, but it should be also of interest to further discuss the potential link between TERT splice variants and CCA immunity. In other words, how these splice variants could modulate the tumor microenvironment, and/or affect the tumor sensitivity to the antitumor immune response (immune evasion and/or immune suppression).

We thank the reviewer for insightful comments regarding immune mechanisms and splice-variant biology. We have expanded the splice variant section to explicitly discuss potential links between rs10069690-associated splicing and tumor immune modulation, while clearly acknowledging that such mechanisms were not directly assessed in our cohort.

In the Discussion, p. 15-16 we now acknowledge the potential for splice variants (e.g., INS1b) to influence immune modulation, that we did not perform splice-variant-specific immune profiling, that effects on immune evasion or suppression remain speculative and require future study:

“However, isoform expression is highly cell-type specific, and leukocyte telomere length has not consistently differed by genotype (28)(29), suggesting that systemic measurements may not reflect tissue-specific effects. Importantly, we did not assess TERT splice variant expression in tumor tissue or immune cell subsets in our cohort. Therefore, it remains unclear whether rs10069690-associated splicing patterns modulate the tumor microenvironment, checkpoint expression, or influence antitumor immune responses in CCA. Dedicated functional and spatially resolved studies will be required to clarify these mechanisms.” (ll. 436-445).

2. TERT splicing analysis with immune profiling could reveal novel therapeutic targets. Are there any results that could be explored in the used databases or on the studied cohort to identify a potential impact of TERT splice variants on immune checkpoint expressions?

The available bulk RNA-seq datasets did not provide reliable isoform-level quantification for TERT splice variants, precluding robust correlation analyses between specific splice isoforms and immune checkpoint gene expression. We clarified that bulk RNA-seq lacks splice-level resolution (ll. 493-495) and that available datasets did not permit robust splice-variant-specific correlation with immune checkpoint genes (ll. 436-445).

We believe that these revisions clarify the exploratory nature of the findings, improve methodological transparency, and appropriately contextualize the results within the current literature.

VERSION 2 - REVIEW

**REVIEWER NAME**	***Wang, Xinwei***
**REVIEWER AFFILIATION**	N/A
**REVIEWER CONFLICT OF INTEREST**	No

AI DISCLOSURE	No, I have not used AI
DATE REVIEW RETURNED	05-Mar-2026

GENERAL COMMENTS	The authors have revised the discussion and limitations to acknowledge several concerns raised in the initial review, which is appreciated. However, the central issue remains unresolved. The manuscript reports discordant findings in which the same allele appears protective for the development of intrahepatic CCA but is associated with worse survival after resection. Survival analysis in this context may reflect risk of relapse or disease progression after surgery and should not be interpreted as an independent biological feature unless validated. The opposing directions of these associations therefore raise concerns regarding the validity and biological plausibility of the findings, and at present the results should only be considered preliminary.<br><br>Importantly, the Abstract still presents the findings too definitively rather than preliminary. For example, the statement “While the TERT T-allele confers protection from intrahepatic CCA in the general population, it is associated with dismal outcomes in patients with manifest disease” is misleading given the exploratory nature of the analysis, the lack of multiple-testing correction, and the absence of independent validation. The abstract should explicitly acknowledge that these findings are exploratory and hypothesis-generating to avoid overstating the strength of the evidence.

REVIEWER NAME	***Lafdil, Fouad***
REVIEWER AFFILIATION	INSERM, CRSA
REVIEWER CONFLICT OF INTEREST	No
AI DISCLOSURE	No, I have not used AI
DATE REVIEW RETURNED	18-Mar-2026

GENERAL COMMENTS	The authors brought a modification on the addressed message of the manuscript accordingly. Some minor correction are still needed:<br>P45-46 of 90: re-write the sentence containing twice "while" in the same sentence... "While rs10069690:T has been... while mitigating the risk"<br>P47: line 447: please correct "This is consistent with <a> potentially" instead of "This is consistent with <an> potentially"

VERSION 2 – AUTHOR RESPONSE

Reviewer: 1

Comments to the Author

The authors have revised the discussion and limitations to acknowledge several concerns raised in the initial review, which is appreciated. However, the central issue remains unresolved. The manuscript reports discordant findings in which the same allele appears protective for the development of intrahepatic CCA but is associated with worse survival after resection. Survival analysis in this context may reflect risk of relapse or disease progression after surgery and should not be interpreted as an independent biological feature unless validated. The opposing directions of these associations therefore raise concerns regarding the validity and biological plausibility of the findings, and at present the results should only be considered preliminary.

Importantly, the Abstract still presents the findings too definitively rather than preliminary. For example, the statement “While the TERT T-allele confers protection from intrahepatic CCA in the general

population, it is associated with dismal outcomes in patients with manifest disease" is misleading given the exploratory nature of the analysis, the lack of multiple-testing correction, and the absence of independent validation. The abstract should explicitly acknowledge that these findings are exploratory and hypothesis-generating to avoid overstating the strength of the evidence.

We thank the reviewer for their time and effort in revising our manuscript. We have revised the abstract to more clearly reflect the exploratory and preliminary nature of our findings.

We removed causal implications, (e.g. changed "confers protection" to "associated with" [l. 62], and changed "identify" to "explore" [l. 37]), highlighted that the results of our analyses are exploratory (see l. 48), and changed the phrasing of the RNA and proteomics analyses to a more cautious tone (e.g. "signatures consistent with", l. 55).

We further rephrased the conclusions section of the abstract to a more exploratory framing of our findings:
"These exploratory, hypothesis-generating findings suggest that the TERT rs10069690 variant may be associated with intrahepatic CCA risk and clinical outcomes, as well as with immune- related and proliferative pathways. The observed context-dependent associations warrant independent validation and further functional investigation." (ll. 62 ff.).

Furthermore, throughout the manuscript, we have now additionally systematically revised the wording to avoid causal interpretations and to emphasize the exploratory nature of the analyses, for example in the Introduction and in the Graphical Abstract (Figure 5).

Reviewer: 2

Comments to the Author
The authors brought a modification on the addressed message of the manuscript accordingly.
Some minor correction are still needed:
P45-46 of 90: re-write the sentence containing twice "while" in the same sentence... "While rs10069690:T has been... while mitigating the risk"
P47: line 447: please correct "This is consistent with potentially" instead of "This is consistent with potentially"

Thank you for your effort in reviewing the manuscript.

We have revised the sentence to avoid double use of “while”: “Although rs10069690:T has been associated with increased risk of several malignancies, it has been linked to reduced risk in only very few malignancies, such as pancreatic cancer, which shares developmental similarities with CCA.[28] We likewise observed an association with reduced iCCA risk in our exploratory UK Biobank analysis.” (ll. 463-467).
We also corrected as follows: “A higher Treg signature was noted in TERT-high iCCA, potentially reflecting the abundance of these immunosuppressive lymphoid populations and an immunosuppressive tumor microenvironment.” (ll. 512-515).

VERSION 3 - REVIEW

**REVIEWER NAME**	***Wang, Xinwei***
**REVIEWER AFFILIATION**	N/A
**REVIEWER CONFLICT OF INTEREST**	No
**AI DISCLOSURE**	No, I have not used AI
**DATE REVIEW RETURNED**	24-Mar-2026

**GENERAL COMMENTS**	The authors have addressed my comments and the revisions are acceptable.

**REVIEWER NAME**	***Lafdil, Fouad***
**REVIEWER AFFILIATION**	INSERM, CRSA
**REVIEWER CONFLICT OF INTEREST**	No
**AI DISCLOSURE**	No, I have not used AI
**DATE REVIEW RETURNED**	01-Apr-2026

**GENERAL COMMENTS**	The authors addressed the raised points accordingly.
